# Supplementary material for: Prevalence and associated factors of last dental visit and teeth cleaning frequency in Bangladesh, Bhutan, and Nepal: Findings from nationally representative surveys
Source: PLOS Glob Public Health. 2024 Jul 19;4(7):e0003511. doi: 10.1371/journal.pgph.0003511 (PMC11259307; doi:10.1371/journal.pgph.0003511)
Supplement: S1 Table — (DOCX) [file pgph.0003511.s001.docx]

**S1 Table: Distribution of the respondents regarding the materials used to clean teeth in Bangladesh and Nepal***

| **Material Used** | **Bangladesh** | **Nepal** |
| --- | --- | --- |
|  | **% (95% CI)** | **% (95% CI)** |
| Toothpaste | 56.76 (54.22-59.26) | 85.71 (80.57-89.67) |
| Toothbrush | 65.33 (62.97-67.61) | 96.45 (94.49-97.73) |
| Wooden toothpick | 4.82 (3.95-5.88) | 12.71 (9.38-17.00) |
| Plastic toothpick | 3.68 (2.57-5.26) | 2.30 (0.97-5.36) |
| Dental floss | 0.33 (0.19-0.57) | 1.65 (0.49-5.40) |
| Charcoal | 22.16 (19.98-24.50) | 1.30 (0.88-1.92) |
| Chewstick/miswak | 15.65 (14.21-17.20) | 3.44 (2.28-5.15) |

*CI: Confidence Interval*

**The data from Bhutan was unavailable*
