# Supplementary material for: Prevalence and associated factors of last dental visit and teeth cleaning frequency in Bangladesh, Bhutan, and Nepal: Findings from nationally representative surveys
Source: PLOS Glob Public Health. 2024 Jul 19;4(7):e0003511. doi: 10.1371/journal.pgph.0003511 (PMC11259307; doi:10.1371/journal.pgph.0003511)
Supplement: S2 Table — (DOCX) [file pgph.0003511.s002.docx]

**S2 Table: Distribution of the respondents regarding the reason for visiting a dentist in Bangladesh and Nepal***

| **Reason** | **Bangladesh** | **Nepal** |
| --- | --- | --- |
|  | **% (95% CI)** | **% (95% CI)** |
| Consultation / advice | 2.14 (1.32-3.44) | 4.63 (2.31-9.08) |
| Pain or trouble with teeth, gums or mouth | 83.71 (80.60-86.40) | 88.10 (80.67-92.92) |
| Treatment / Follow-up treatment | 12.70 (10.37-15.46) | 4.06 (2.03-7.97) |
| Routine check-up treatment | 1.07 (0.55-2.08) | 2.51 (0.82-7.44) |
| Other | 0.39 (0.20-0.76) | 0.70 (0.16-3.08) |

*CI: Confidence Interval*

**The data from Bhutan was unavailable*
