# Supplementary material for: Prevalence and associated factors of last dental visit and teeth cleaning frequency in Bangladesh, Bhutan, and Nepal: Findings from nationally representative surveys
Source: PLOS Glob Public Health. 2024 Jul 19;4(7):e0003511. doi: 10.1371/journal.pgph.0003511 (PMC11259307; doi:10.1371/journal.pgph.0003511)
Supplement: S3 Table — (DOCX) [file pgph.0003511.s003.docx]

**S3 Table: Crude and adjusted prevalence ratios and odds ratio for the factors associated with cleaning teeth at least once a day in Bangladesh**

| **Characteristics** | **COR (95% CI)** | **P-value** | **CPR (95% CI)** | **P-value** | **AOR (95% CI)** | **P-value** | **APR (95% CI)** | **P-value** |
| --- | --- | --- | --- | --- | --- | --- | --- | --- |
| **Age Group (in years)** |  |  |  |  |  |  |  |  |
| 18–29 | Ref |  | Ref |  | Ref |  | Ref |  |
| 30-49 | 0.78 (0.35-1.78) | 0.563 | 1.00 (0.99-1.00) | 0.081 | 1.59 (0.64-3.92) | 0.315 | 1.00 (1.00-1.01) | 0.630 |
| 50-69 | 0.24 (0.11-0.54) | 0.001 | 0.98 (0.97-0.99) | 0.001 | 0.68 (0.27-1.73) | 0.423 | 0.98 (0.98-1.00) | 0.060 |
| **Gender** |  |  |  |  |  |  |  |  |
| Male | Ref |  | Ref |  | Ref |  | Ref |  |
| Female | 4.80 (2.52-9.13) | <0.001 | 1.01 (1.01-1.02) | <0.001 | 1.44 (0.48-4.28) | 0.512 | 1.01 (0.99-1.01) | 0.940 |
| **Highest Educational Attainment** |  |  |  |  |  |  |  |  |
| No Formal Education | Ref |  | Ref |  | Ref |  | Ref |  |
| Up to primary | 2.78 (1.57-4.91) | <0.001 | 1.01 (1.00-1.02) | 0.001 | 2.33 (1.25-4.35) | 0.008 | 1.01 (1.00-1.02) | 0.040 |
| Up to secondary | 5.66 (1.96-16.37) | 0.001 | 1.01 (1.01-1.02) | 0.001 | 4.52 (1.48-13.77) | 0.008 | 1.01 (1.00-1.02) | 0.060 |
| College and higher | 1.00 (-) |  | 1.02 (1.01-1.02) | <0.001 | 1.00 (-) |  | 1.02 (1.00-1.02) | <0.001 |
| **Marital Status** |  |  |  |  |  |  |  |  |
| Never married | Ref |  | Ref |  | Ref |  | Ref |  |
| Currently married | 0.22 (0.03-1.62) | 0.138 | 0.99 (0.99-1.00) | 0.091 | 0.36 (0.04-3.03) | 0.344 | 0.99 (0.99-1.01) | 0.810 |
| Divorced/widowed/separated | 0.77 (0.05-12.73) | 0.856 | 1.00 (1.00-1.01) | 0.357 | 1.25 (0.06-27.52) | 0.887 | 1.00 (1.00-1.03) | 0.060 |
| **Smoking Status** |  |  |  |  |  |  |  |  |
| Never Smoker | Ref |  | Ref |  | Ref |  | Ref |  |
| Current Smoker | 0.14 (0.08-0.27) | <0.001 | 0.98 (0.97-0.99) | <0.001 | 0.22 (0.08-0.60) | 0.003 | 0.98 (0.98-0.99) | <0.001 |
| Former Smoker | 0.11 (0.05-0.25) | <0.001 | 0.98 (0.95-1.00) | 0.023 | 0.20 (0.06-0.61) | 0.005 | 0.98 (0.96-1.00) | 0.070 |
| **Ever Alcohol Consumption** |  |  |  |  |  |  |  |  |
| Yes | Ref |  | Ref |  | Ref |  | Ref |  |
| No | 1.51 (0.65-3.49) | 0.338 | 1.01 (0.99-1.03) | 0.327 | 0.73 (0.28-1.90) | 0.515 | 1.01 (0.98-1.02) | 0.720 |
| **Dental Visit** |  |  |  |  |  |  |  |  |
| Less than 6 months | Ref |  | Ref |  | Ref |  | Ref |  |
| 6-12 months | 4.43 (0.52-37.85) | 0.173 | 1.00 (1.00-1.01) | 0.115 | 5.39 (0.58-50.37) | 0.139 | 1.00 (1.00-1.01) | 0.520 |
| More than 12 months | 1.01 (0.37-2.79) | 0.985 | 0.99 (0.98-1.00) | 0.088 | 1.18 (0.40-3.46) | 0.767 | 0.99 (0.98-1.00) | 0.070 |
| Never visited | 1.28 (0.53-3.13) | 0.582 | 1.00 (0.99-1.00) | 0.421 | 1.69 (0.65-4.37) | 0.277 | 1.00 (0.99-1.00) | 0.250 |

*AOR: Adjusted Odds Ratio; APR: Adjusted Prevalence Ratio; CI: Confidence Interval; COR: Crude Odds Ratio; CPR: Crude Prevalence Ratio*
