# Supplementary material for: Prevalence and associated factors of last dental visit and teeth cleaning frequency in Bangladesh, Bhutan, and Nepal: Findings from nationally representative surveys
Source: PLOS Glob Public Health. 2024 Jul 19;4(7):e0003511. doi: 10.1371/journal.pgph.0003511 (PMC11259307; doi:10.1371/journal.pgph.0003511)
Supplement: S4 Table — (DOCX) [file pgph.0003511.s004.docx]

**S4 Table: Crude and adjusted prevalence ratios and odds ratio for the factors associated with cleaning teeth at least once a day in Bhutan**

| **Characteristics** | **COR (95% CI)** | **P-value** | **CPR (95% CI)** | **P-value** | **AOR (95% CI)** | **P-value** | **APR (95% CI)** | **P-value** |
| --- | --- | --- | --- | --- | --- | --- | --- | --- |
| **Age Group (in years)** |  |  |  |  |  |  |  |  |
| 18–29 | Ref |  | Ref |  | Ref |  | Ref |  |
| 30-49 | 0.44 (0.27-0.71) | 0.001 | 0.96 (0.93-0.99) | 0.006 | 0.61 (0.36-1.04) | 0.068 | 1.00 (0.96-1.04) | 0.953 |
| 50-69 | 0.12 (0.07-0.19) | <0.001 | 0.77 (0.72-0.83) | <0.001 | 0.21 (0.12-0.37) | <0.001 | 0.82 (0.76-0.89) | <0.001 |
| **Gender** |  |  |  |  |  |  |  |  |
| Male | Ref |  | Ref |  | Ref |  | Ref |  |
| Female | 1.68 (1.30-2.18) | <0.001 | 1.04 (1.01-1.06) | 0.014 | 1.69 (1.27-2.25) | <0.001 | 1.05 (1.02-1.09) | 0.004 |
| **Highest Educational Attainment** |  |  |  |  |  |  |  |  |
| No Formal Education | Ref |  | Ref |  | Ref |  | Ref |  |
| Up to primary | 2.16 (1.46-3.21) | <0.001 | 1.08 (1.02-1.13) | 0.004 | 1.81 (1.19-2.74) | 0.005 | 1.04 (0.99-1.10) | 0.087 |
| Up to secondary | 9.78 (4.93-19.40) | <0.001 | 1.17 (1.13-1.22) | <0.001 | 7.67 (3.72-15.80) | <0.001 | 1.12 (1.07-1.16) | <0.001 |
| College and higher | 6.53 (2.02-21.07) | 0.002 | 1.16 (1.11-1.21) | <0.001 | 6.12 (1.84-20.39) | 0.003 | 1.12 (1.07-1.18) | <0.001 |
| **Marital Status** |  |  |  |  |  |  |  |  |
| Never married | Ref |  | Ref |  | Ref |  | Ref |  |
| Currently married | 0.71 (0.42-1.18) | 0.186 | 0.93 (0.90-0.96) | <0.001 | 2.08 (1.14-3.80) | 0.018 | 1.01 (0.98-1.05) | 0.396 |
| Divorced/widowed/separated | 0.54 (0.29-0.99) | 0.048 | 0.89 (0.84-0.94) | <0.001 | 1.81 (0.89-3.68) | 0.099 | 1.01 (0.95-1.07) | 0.782 |
| **Smoking Status** |  |  |  |  |  |  |  |  |
| Never Smoker | Ref |  | Ref |  | Ref |  | Ref |  |
| Current Smoker | 2.03 (0.96-4.29) | 0.063 | 1.08 (1.05-1.11) | <0.001 | 1.56 (0.70-3.48) | 0.274 | 1.04 (1.01-1.08) | 0.015 |
| Former Smoker | 0.83 (0.58-1.17) | 0.288 | 1.02 (0.98-1.05) | 0.327 | 1.02 (0.70-1.48) | 0.935 | 1.02 (0.99-1.06) | 0.207 |
| **Ever Alcohol Consumption** |  |  |  |  |  |  |  |  |
| Yes | Ref |  | Ref |  | Ref |  | Ref |  |
| No | 1.82 (1.36-2.43) | <0.001 | 1.05 (1.01-1.09) | 0.023 | 1.63 (1.20-2.21) | 0.002 | 1.04 (1.00-1.08) | 0.049 |
| **Dental Visit** |  |  |  |  |  |  |  |  |
| Less than 6 months | Ref |  | Ref |  | Ref |  | Ref |  |
| 6-12 months | 1.00 (-) |  | 1.07 (0.95-1.22) | 0.268 | 1.00 (-) |  | 1.07 (0.84-1.35) | 0.596 |
| More than 12 months | 0.64 (0.06-7.47) | 0.724 | 0.85 (0.69-1.05) | 0.131 | 1.10 (0.09-13.92) | 0.943 | 0.90 (0.74-1.08) | 0.247 |
| Never visited | 1.57 (0.17-14.68) | 0.691 | 0.98 (0.86-1.11) | 0.715 | 2.45 (0.25-24.13) | 0.442 | 0.99 (0.84-1.17) | 0.907 |

*AOR: Adjusted Odds Ratio; APR: Adjusted Prevalence Ratio; CI: Confidence Interval; COR: Crude Odds Ratio; CPR: Crude Prevalence Ratio*
