# Supplementary material for: Prevalence and associated factors of last dental visit and teeth cleaning frequency in Bangladesh, Bhutan, and Nepal: Findings from nationally representative surveys
Source: PLOS Glob Public Health. 2024 Jul 19;4(7):e0003511. doi: 10.1371/journal.pgph.0003511 (PMC11259307; doi:10.1371/journal.pgph.0003511)
Supplement: S5 Table — (DOCX) [file pgph.0003511.s005.docx]

**S5 Table: Crude and adjusted prevalence ratios and odds ratio for the factors associated with cleaning teeth at least once a day in Nepal**

| **Characteristics** | **COR (95% CI)** | **P-value** | **CPR (95% CI)** | **P-value** | **AOR (95% CI)** | **P-value** | **APR (95% CI)** | **P-value** |
| --- | --- | --- | --- | --- | --- | --- | --- | --- |
| **Age Group (in years)** |  |  |  |  |  |  |  |  |
| 18–29 | Ref |  | Ref |  | Ref |  | Ref |  |
| 30-49 | 0.30 (0.21-0.41) | <0.001 | 0.93 (0.89-0.97) | <0.001 | 0.50 (0.35-0.72) | <0.001 | 0.95 (0.91-0.99) | 0.021 |
| 50-69 | 0.09 (0.06-0.12) | <0.001 | 0.83 (0.79-0.87) | <0.001 | 0.23 (0.15-0.34) | <0.001 | 0.87 (0.82-0.93) | <0.001 |
| **Gender** |  |  |  |  |  |  |  |  |
| Male | Ref |  | Ref |  | Ref |  | Ref |  |
| Female | 1.15 (0.95-1.39) | 0.157 | 1.00 (0.97-1.03) | 0.886 | 0.93 (0.72-1.20) | 0.577 | 0.98 (0.95-1.02) | 0.393 |
| **Highest Educational Attainment** |  |  |  |  |  |  |  |  |
| No Formal Education | Ref |  | Ref |  | Ref |  | Ref |  |
| Up to primary | 2.72 (2.16-3.44) | <0.001 | 1.10 (1.05-1.15) | <0.001 | 1.99 (1.52-2.60) | <0.001 | 1.05 (1.01-1.11) | 0.028 |
| Up to secondary | 7.27 (5.30-9.97) | <0.001 | 1.14 (1.08-1.20) | <0.001 | 3.40 (2.35-4.92) | <0.001 | 1.08 (1.01-1.13) | 0.048 |
| College and higher | 6.43 (2.82-14.68) | <0.001 | 1.19 (1.13-1.25) | <0.001 | 3.16 (1.32-7.57) | 0.01 | 1.13 (1.04-1.17) | 0.001 |
| **Marital Status** |  |  |  |  |  |  |  |  |
| Never married | Ref |  | Ref |  | Ref |  | Ref |  |
| Currently married | 0.37 (0.22-0.63) | <0.001 | 0.93 (0.90-0.96) | <0.001 | 1.30 (0.71-2.37) | 0.39 | 0.90 (0.97-1.04) | 0.823 |
| Divorced/widowed/separated | 0.09 (0.05-0.16) | <0.001 | 0.78 (0.69-0.87) | <0.001 | 0.65 (0.32-1.30) | 0.219 | 0.69 (0.83-1.03) | 0.164 |
| **Smoking Status** |  |  |  |  |  |  |  |  |
| Never Smoker | Ref |  | Ref |  | Ref |  | Ref |  |
| Current Smoker | 0.29 (0.23-0.36) | <0.001 | 0.90 (0.86-0.94) | <0.001 | 0.49 (0.38-0.63) | <0.001 | 0.86 (0.89-0.97) | 0.002 |
| Former Smoker | 0.34 (0.25-0.46) | <0.001 | 0.92 (0.86-0.99) | 0.020 | 0.66 (0.47-0.94) | 0.022 | 0.86 (0.92-1.05) | 0.675 |
| **Ever Alcohol Consumption** |  |  |  |  |  |  |  |  |
| Yes | Ref |  | Ref |  | Ref |  | Ref |  |
| No | 1.94 (1.57-2.40) | <0.001 | 1.05 (1.01-1.09) | 0.019 | 1.52 (1.17-1.98) | 0.002 | 1.01 (0.98-1.07) | 0.367 |
| **Dental Visit** |  |  |  |  |  |  |  |  |
| Less than 6 months | Ref |  | Ref |  | Ref |  | Ref |  |
| 6-12 months | 1.14 (0.45-2.87) | 0.780 | 1.02 (0.86-1.22) | 0.788 | 0.99 (0.37-2.66) | 0.977 | 0.86 (0.87-1.20) | 0.789 |
| More than 12 months | 1.17 (0.57-2.41) | 0.670 | 1.02 (0.90-1.16) | 0.698 | 1.18 (0.54-2.56) | 0.683 | 0.90 (0.92-1.16) | 0.587 |
| Never visited | 1.65 (0.92-2.97) | 0.092 | 1.05 (0.95-1.17) | 0.322 | 1.37 (0.73-2.57) | 0.33 | 0.95 (0.92-1.13) | 0.692 |

*AOR: Adjusted Odds Ratio; APR: Adjusted Prevalence Ratio; CI: Confidence Interval; COR: Crude Odds Ratio; CPR: Crude Prevalence Ratio*
