# Supplementary material for: Prevalence and associated factors of last dental visit and teeth cleaning frequency in Bangladesh, Bhutan, and Nepal: Findings from nationally representative surveys
Source: PLOS Glob Public Health. 2024 Jul 19;4(7):e0003511. doi: 10.1371/journal.pgph.0003511 (PMC11259307; doi:10.1371/journal.pgph.0003511)
Supplement: S6 Table — (DOCX) [file pgph.0003511.s006.docx]

**S6 Table: Crude and adjusted prevalence ratios and odds ratio for the factors associated with cleaning teeth at least twice a day in Bangladesh**

| **Characteristics** | **COR (95% CI)** | **P-value** | **CPR (95% CI)** | **P-value** | **AOR (95% CI)** | **P-value** | **APR (95% CI)** | **P-value** |
| --- | --- | --- | --- | --- | --- | --- | --- | --- |
| **Age Group (in years)** |  |  |  |  |  |  |  |  |
| 18–29 | Ref |  | Ref |  | Ref |  | Ref |  |
| 30-49 | 1.22 (1.09-1.37) | 0.001 | 1.13 (1.03-1.23) | 0.010 | 1.42 (1.24-1.62) | <0.001 | 1.17 (1.06-1.29) | <0.001 |
| 50-69 | 1.16 (1.01-1.33) | 0.040 | 1.17 (1.05-1.30) | 0.010 | 1.56 (1.32-1.85) | <0.001 | 1.23 (1.09-1.39) | <0.001 |
| **Gender** |  |  |  |  |  |  |  |  |
| Male | Ref |  | Ref |  | Ref |  | Ref |  |
| Female | 2.42 (2.20-2.66) | <0.001 | 1.49 (1.37-1.63) | <0.001 | 2.56 (2.22-2.96) | <0.001 | 1.55 (1.37-1.75) | <0.001 |
| **Highest Educational Attainment** |  |  |  |  |  |  |  |  |
| No Formal Education | Ref |  | Ref |  | Ref |  | Ref |  |
| Up to primary | 1.01 (0.90-1.13) | 0.879 | 0.95 (0.86-1.05) | 0.330 | 1.05 (0.92-1.18) | 0.481 | 1.03 (0.93-1.13) | 0.590 |
| Up to secondary | 1.19 (1.03-1.38) | 0.022 | 1.03 (0.91-1.17) | 0.680 | 1.47 (1.25-1.73) | <0.001 | 1.20 (1.06-1.36) | <0.001 |
| College and higher | 1.90 (1.53-2.36) | <0.001 | 1.29 (1.11-1.49) | <0.001 | 2.48 (1.97-3.13) | <0.001 | 1.53 (1.32-1.77) | <0.001 |
| **Marital Status** |  |  |  |  |  |  |  |  |
| Never married | Ref |  | Ref |  | Ref |  | Ref |  |
| Currently married | 1.35 (1.11-1.64) | 0.002 | 0.75 (0.67-0.84) | <0.001 | 0.94 (0.75-1.18) | 0.612 | 1.00 (0.84-1.20) | 0.960 |
| Divorced/widowed/separated | 2.29 (1.71-3.06) | <0.001 | 0.85 (0.74-0.97) | 0.020 | 1.01 (0.72-1.42) | 0.937 | 1.11 (0.87-1.41) | 0.400 |
| **Smoking Status** |  |  |  |  |  |  |  |  |
| Never Smoker | Ref |  | Ref |  | Ref |  | Ref |  |
| Current Smoker | 0.46 (0.41-0.52) | <0.001 | 1.18 (1.01-1.38) | 0.040 | 0.93 (0.79-1.09) | 0.383 | 1.05 (0.91-1.21) | 0.480 |
| Former Smoker | 0.58 (0.49-0.70) | <0.001 | 1.60 (1.30-1.98) | <0.001 | 1.06 (0.85-1.31) | 0.603 | 1.09 (0.93-1.28) | 0.290 |
| **Ever Alcohol Consumption** |  |  |  |  |  |  |  |  |
| Yes | Ref |  | Ref |  | Ref |  | Ref |  |
| No | 1.76 (1.47-2.12) | <0.001 | 1.43 (1.20-1.70) | <0.001 | 1.03 (0.84-1.26) | 0.766 | 1.13 (0.95-1.34) | 0.160 |
| **Dental Visit** |  |  |  |  |  |  |  |  |
| Less than 6 months | Ref |  | Ref |  | Ref |  | Ref |  |
| 6-12 months | 0.87 (0.67-1.12) | 0.277 | 1.04 (0.84-1.28) | 0.740 | 0.85 (0.65-1.10) | 0.220 | 1.01 (0.82-1.26) | 0.900 |
| More than 12 months | 0.92 (0.75-1.13) | 0.426 | 1.10 (0.95-1.27) | 0.220 | 0.90 (0.73-1.11) | 0.309 | 1.07 (0.92-1.24) | 0.360 |
| Never visited | 0.83 (0.70-0.99) | 0.044 | 1.03 (0.90-1.17) | 0.700 | 0.88 (0.73-1.05) | 0.153 | 1.05 (0.92-1.20) | 0.490 |

*AOR: Adjusted Odds Ratio; APR: Adjusted Prevalence Ratio; CI: Confidence Interval; COR: Crude Odds Ratio; CPR: Crude Prevalence Ratio*
