# Supplementary material for: Prevalence and associated factors of last dental visit and teeth cleaning frequency in Bangladesh, Bhutan, and Nepal: Findings from nationally representative surveys
Source: PLOS Glob Public Health. 2024 Jul 19;4(7):e0003511. doi: 10.1371/journal.pgph.0003511 (PMC11259307; doi:10.1371/journal.pgph.0003511)
Supplement: S7 Table — (DOCX) [file pgph.0003511.s007.docx]

**S7 Table: Crude and adjusted prevalence ratios and odds ratio for the factors associated with cleaning teeth at least twice a day in Bhutan**

| **Characteristics** | **COR (95% CI)** | **P-value** | **CPR (95% CI)** | **P-value** | **AOR (95% CI)** | **P-value** | **APR (95% CI)** | **P-value** |
| --- | --- | --- | --- | --- | --- | --- | --- | --- |
| **Age Group (in years)** |  |  |  |  |  |  |  |  |
| 18–29 | Ref |  | Ref |  | Ref |  | Ref |  |
| 30-49 | 0.68 (0.55-0.86) | 0.001 | 0.78 (0.64-0.96) | 0.019 | 1.05 (0.81-1.36) | 0.732 | 1.21 (0.97-1.51) | 0.089 |
| 50-69 | 0.39 (0.29-0.52) | <0.001 | 0.51 (0.37-0.68) | <0.001 | 0.77 (0.54-1.09) | 0.134 | 1.01 (0.71-1.44) | 0.958 |
| **Gender** |  |  |  |  |  |  |  |  |
| Male | Ref |  | Ref |  | Ref |  | Ref |  |
| Female | 1.38 (1.14-1.68) | 0.001 | 1.37 (1.15-1.63) | 0.001 | 1.66 (1.33-2.08) | <0.001 | 1.64 (1.34-2.01) | <0.001 |
| **Highest Educational Attainment** |  |  |  |  |  |  |  |  |
| No Formal Education | Ref |  | Ref |  | Ref |  | Ref |  |
| Up to primary | 1.60 (1.22-2.10) | 0.001 | 1.40 (1.06-1.84) | 0.018 | 1.64 (1.24-2.18) | 0.001 | 1.45 (1.10-1.91) | 0.009 |
| Up to secondary | 3.17 (2.51-4.00) | <0.001 | 2.29 (1.78-2.93) | <0.001 | 3.00 (2.30-3.91) | <0.001 | 2.11 (1.50-2.96) | <0.001 |
| College and higher | 3.90 (2.62-5.80) | <0.001 | 2.73 (2.07-3.60) | <0.001 | 4.41 (2.90-6.72) | <0.001 | 2.98 (2.21-4.02) | <0.001 |
| **Marital Status** |  |  |  |  |  |  |  |  |
| Never married | Ref |  | Ref |  | Ref |  | Ref |  |
| Currently married | 0.51 (0.38-0.68) | <0.001 | 0.58 (0.45-0.73) | <0.001 | 0.80 (0.57-1.12) | 0.188 | 0.73 (0.54-0.99) | 0.046 |
| Divorced/widowed/separated | 0.47 (0.31-0.72) | <0.001 | 0.48 (0.33-0.69) | <0.001 | 0.79 (0.49-1.27) | 0.329 | 0.63 (0.40-0.98) | 0.040 |
| **Smoking Status** |  |  |  |  |  |  |  |  |
| Never Smoker | Ref |  | Ref |  | Ref |  | Ref |  |
| Current Smoker | 1.19 (0.83-1.72) | 0.343 | 1.23 (0.84-1.79) | 0.289 | 1.02 (0.69-1.52) | 0.917 | 1.15 (0.78-1.69) | 0.478 |
| Former Smoker | 1.22 (0.95-1.57) | 0.120 | 1.21 (0.93-1.58) | 0.161 | 1.37 (1.04-1.80) | 0.023 | 1.31 (1.01-1.71) | 0.046 |
| **Ever Alcohol Consumption** |  |  |  |  |  |  |  |  |
| Yes | Ref |  | Ref |  | Ref |  | Ref |  |
| No | 1.50 (1.24-1.83) | <0.001 | 1.38 (1.10-1.73) | 0.006 | 1.48 (1.20-1.82) | <0.001 | 1.31 (1.06-1.61) | 0.012 |
| **Dental Visit** |  |  |  |  |  |  |  |  |
| Less than 6 months | Ref |  | Ref |  | Ref |  | Ref |  |
| 6-12 months | 3.18 (0.21-47.75) | 0.402 | 1.32 (0.64-2.73) | 0.442 | 3.54 (0.20-63.06) | 0.389 | 1.41 (0.40-5.04) | 0.590 |
| More than 12 months | 0.50 (0.06-4.33) | 0.528 | 0.60 (0.27-1.33) | 0.206 | 0.70 (0.08-6.41) | 0.752 | 0.84 (0.33-2.11) | 0.708 |
| Never visited | 0.79 (0.13-4.71) | 0.798 | 0.48 (0.16-1.43) | 0.185 | 0.87 (0.14-5.37) | 0.878 | 0.50 (0.13-1.93) | 0.313 |

*AOR: Adjusted Odds Ratio; APR: Adjusted Prevalence Ratio; CI: Confidence Interval; COR: Crude Odds Ratio; CPR: Crude Prevalence Ratio*
