# Supplementary material for: Prevalence and associated factors of last dental visit and teeth cleaning frequency in Bangladesh, Bhutan, and Nepal: Findings from nationally representative surveys
Source: PLOS Glob Public Health. 2024 Jul 19;4(7):e0003511. doi: 10.1371/journal.pgph.0003511 (PMC11259307; doi:10.1371/journal.pgph.0003511)
Supplement: S8 Table — (DOCX) [file pgph.0003511.s008.docx]

**S8 Table: Crude and adjusted prevalence ratios and odds ratio for the factors associated with cleaning teeth at least twice a day in Nepal**

| **Characteristics** | **COR (95% CI)** | **P-value** | **CPR (95% CI)** | **P-value** | **AOR (95% CI)** | **P-value** | **APR (95% CI)** | **P-value** |
| --- | --- | --- | --- | --- | --- | --- | --- | --- |
| **Age Group (in years)** |  |  |  |  |  |  |  |  |
| 18–29 | Ref |  | Ref |  | Ref |  | Ref |  |
| 30-49 | 0.52 (0.40-0.69) | <0.001 | 0.55 (0.38-0.81) | 0.002 | 0.74 (0.54-1.01) | 0.06 | 0.76 (0.46-1.26) | 0.285 |
| 50-69 | 0.34 (0.24-0.48) | <0.001 | 0.37 (0.25-0.53) | <0.001 | 0.72 (0.47-1.11) | 0.135 | 0.69 (0.38-1.23) | 0.208 |
| **Gender** |  |  |  |  |  |  |  |  |
| Male | Ref |  | Ref |  | Ref |  | Ref |  |
| Female | 1.38 (1.07-1.79) | 0.015 | 1.27 (0.87-1.86) | 0.209 | 1.65 (1.20-2.27) | 0.002 | 1.59 (1.11-2.29) | 0.012 |
| **Highest Educational Attainment** |  |  |  |  |  |  |  |  |
| No Formal Education | Ref |  | Ref |  | Ref |  | Ref |  |
| Up to primary | 1.73 (1.22-2.45) | 0.002 | 1.26 (0.78-2.05) | 0.346 | 1.76 (1.21-2.58) | 0.003 | 1.13 (0.65-1.99) | 0.662 |
| Up to secondary | 3.24 (2.35-4.47) | <0.001 | 2.61 (1.60-4.27) | <0.001 | 3.10 (2.11-4.55) | <0.001 | 2.13 (1.13-4.02) | 0.020 |
| College and higher | 7.09 (4.27-11.76) | <0.001 | 5.44 (2.81-10.53) | <0.001 | 7.10 (4.04-12.45) | <0.001 | 4.27 (1.99-9.18) | <0.001 |
| **Marital Status** |  |  |  |  |  |  |  |  |
| Never married | Ref |  | Ref |  | Ref |  | Ref |  |
| Currently married | 0.49 (0.33-0.74) | 0.001 | 0.48 (0.32-0.73) | 0.001 | 0.85 (0.54-1.34) | 0.488 | 0.72 (0.45-1.15) | 0.170 |
| Divorced/widowed/separated | 0.34 (0.17-0.69) | 0.003 | 0.28 (0.13-0.59) | 0.001 | 0.91 (0.42-1.98) | 0.811 | 0.60 (0.27-1.36) | 0.224 |
| **Smoking Status** |  |  |  |  |  |  |  |  |
| Never Smoker | Ref |  | Ref |  | Ref |  | Ref |  |
| Current Smoker | 0.46 (0.32-0.67) | <0.001 | 0.57 (0.36-0.92) | 0.021 | 0.70 (0.46-1.08) | 0.107 | 0.63 (0.38-1.05) | 0.075 |
| Former Smoker | 0.77 (0.48-1.23) | 0.274 | 0.64 (0.37-1.11) | 0.110 | 1.34 (0.81-2.21) | 0.261 | 0.77 (0.44-1.35) | 0.361 |
| **Ever Alcohol Consumption** |  |  |  |  |  |  |  |  |
| Yes | Ref |  | Ref |  | Ref |  | Ref |  |
| No | 1.35 (1.02-1.78) | 0.034 | 0.92 (0.62-1.36) | 0.671 | 0.96 (0.69-1.34) | 0.798 | 0.59 (0.39-0.90) | 0.014 |
| **Dental Visit** |  |  |  |  |  |  |  |  |
| Less than 6 months | Ref |  | Ref |  | Ref |  | Ref |  |
| 6-12 months | 1.95 (0.69-5.54) | 0.210 | 0.81 (0.25-2.58) | 0.718 | 1.72 (0.59-5.06) | 0.324 | 0.70 (0.23-2.17) | 0.533 |
| More than 12 months | 1.05 (0.43-2.58) | 0.919 | 0.62 (0.16-2.44) | 0.488 | 1.03 (0.41-2.59) | 0.945 | 0.70 (0.18-2.80) | 0.618 |
| Never visited | 0.91 (0.45-1.85) | 0.798 | 0.67 (0.29-1.52) | 0.336 | 0.82 (0.40-1.69) | 0.592 | 0.59 (0.26-1.33) | 0.201 |

*AOR: Adjusted Odds Ratio; APR: Adjusted Prevalence Ratio; CI: Confidence Interval; COR: Crude Odds Ratio; CPR: Crude Prevalence Ratio*
