# Supplementary material for: Prevalence and associated factors of last dental visit and teeth cleaning frequency in Bangladesh, Bhutan, and Nepal: Findings from nationally representative surveys
Source: PLOS Glob Public Health. 2024 Jul 19;4(7):e0003511. doi: 10.1371/journal.pgph.0003511 (PMC11259307; doi:10.1371/journal.pgph.0003511)
Supplement: S9 Table — (DOCX) [file pgph.0003511.s009.docx]

**S9 Table: Crude and adjusted prevalence ratios and odds ratio for the factors associated with visiting a dentist in last six months in Bangladesh**

| **Characteristics** | **COR (95% CI)** | **P-value** | **CPR (95% CI)** | **P-value** | **AOR (95% CI)** | **P-value** | **APR (95% CI)** | **P-value** |
| --- | --- | --- | --- | --- | --- | --- | --- | --- |
| **Age Group (in years)** |  |  |  |  |  |  |  |  |
| 18–29 | Ref |  | Ref |  | Ref |  | Ref |  |
| 30-49 | 1.35 (1.09-1.67) | 0.006 | 1.32 (1.02-1.72) | 0.040 | 1.51 (1.19-1.91) | 0.001 | 1.40 (1.04-1.88) | 0.030 |
| 50-69 | 1.39 (1.08-1.78) | 0.010 | 1.40 (1.02-1.93) | 0.040 | 1.70 (1.28-2.27) | <0.001 | 1.63 (1.11-2.39) | 0.010 |
| **Gender** |  |  |  |  |  |  |  |  |
| Male | Ref |  | Ref |  | Ref |  | Ref |  |
| Female | 1.04 (0.89-1.23) | 0.606 | 0.98 (0.79-1.22) | 0.840 | 1.12 (0.88-1.42) | 0.368 | 1.24 (0.87-1.75) | 0.230 |
| **Highest Educational Attainment** |  |  |  |  |  |  |  |  |
| No Formal Education | Ref |  | Ref |  | Ref |  | Ref |  |
| Up to primary | 1.27 (1.04-1.55) | 0.018 | 1.08 (0.83-1.40) | 0.560 | 1.38 (1.12-1.70) | 0.002 | 1.23 (0.96-1.57) | 0.110 |
| Up to secondary | 1.26 (0.98-1.63) | 0.072 | 0.94 (0.64-1.39) | 0.770 | 1.42 (1.08-1.85) | 0.011 | 1.15 (0.76-1.72) | 0.510 |
| College and higher | 1.41 (1.00-1.97) | 0.047 | 1.16 (0.72-1.87) | 0.540 | 1.51 (1.06-2.14) | 0.022 | 1.38 (0.85-2.26) | 0.200 |
| **Marital Status** |  |  |  |  |  |  |  |  |
| Never married | Ref |  | Ref |  | Ref |  | Ref |  |
| Currently married | 1.08 (0.78-1.52) | 0.638 | 1.16 (0.77-1.77) | 0.480 | 0.85 (0.58-1.24) | 0.390 | 0.88 (0.56-1.41) | 0.610 |
| Divorced/widowed/separated | 1.01 (0.62-1.67) | 0.954 | 0.77 (0.41-1.42) | 0.400 | 0.74 (0.42-1.30) | 0.297 | 0.51 (0.25-1.03) | 0.060 |
| **Smoking Status** |  |  |  |  |  |  |  |  |
| Never Smoker | Ref |  | Ref |  | Ref |  | Ref |  |
| Current Smoker | 0.94 (0.77-1.14) | 0.536 | 1.16 (0.88-1.52) | 0.300 | 0.97 (0.73-1.27) | 0.799 | 1.27 (0.85-1.92) | 0.250 |
| Fomer Smoker | 1.06 (0.78-1.43) | 0.707 | 1.30 (0.87-1.94) | 0.200 | 1.04 (0.73-1.48) | 0.840 | 1.34 (0.84-2.12) | 0.220 |
| **Ever Alcohol Consumption** |  |  |  |  |  |  |  |  |
| Yes | Ref |  | Ref |  | Ref |  | Ref |  |
| No | 0.89 (0.66-1.19) | 0.423 | 1.11 (0.76-1.60) | 0.590 | 0.84 (0.61-1.15) | 0.269 | 1.12 (0.75-1.66) | 0.580 |
| **Teeth Cleaning Frequency** |  |  |  |  |  |  |  |  |
| Once a day | Ref |  | Ref |  | Ref |  | Ref |  |
| Twice a day | 1.15 (0.97-1.36) | 0.104 | 0.93 (0.75-1.16) | 0.530 | 1.10 (0.93-1.31) | 0.257 | 0.91 (0.72-1.14) | 0.410 |
| Infrequent/Never | 1.36 (0.57-3.29) | 0.488 | 0.63 (0.24-1.66) | 0.350 | 1.43 (0.59-3.47) | 0.428 | 0.54 (0.20-1.44) | 0.220 |

*AOR: Adjusted Odds Ratio; APR: Adjusted Prevalence Ratio; CI: Confidence Interval; COR: Crude Odds Ratio; CPR: Crude Prevalence Ratio*
