# Supplementary material for: Prevalence and associated factors of last dental visit and teeth cleaning frequency in Bangladesh, Bhutan, and Nepal: Findings from nationally representative surveys
Source: PLOS Glob Public Health. 2024 Jul 19;4(7):e0003511. doi: 10.1371/journal.pgph.0003511 (PMC11259307; doi:10.1371/journal.pgph.0003511)
Supplement: S10 Table — (DOCX) [file pgph.0003511.s010.docx]

**S10 Table: Crude and adjusted prevalence ratios and odds ratio for the factors associated with visiting a dentist in last six months in Bhutan**

| **Characteristics** | **COR (95% CI)** | **P-value** | **CPR (95% CI)** | **P-value** | **AOR (95% CI)** | **P-value** | **APR (95% CI)** | **P-value** |
| --- | --- | --- | --- | --- | --- | --- | --- | --- |
| **Age Group (in years)** |  |  |  |  |  |  |  |  |
| 18–29 | Ref |  | Ref |  | Ref |  | Ref |  |
| 30-49 | 0.69 (0.56-0.85) | 0.001 | 0.74 (0.57-0.97) | 0.028 | 0.86 (0.12-6.01) | 0.879 | 1.16 (0.87-1.55) | 0.296 |
| 50-69 | 0.64 (0.50-0.83) | 0.001 | 0.69 (0.50-0.96) | 0.030 | 1.00 (-) |  | 0.00 (0.00-0.00) | <0.001 |
| **Gender** |  |  |  |  |  |  |  |  |
| Male | Ref |  | Ref |  | Ref |  | Ref |  |
| Female | 1.36 (1.12-1.64) | 0.002 | 1.39 (1.10-1.75) | 0.006 | 1.19 (0.16-8.99) | 0.866 | 1.18 (0.10-14.29) | 0.894 |
| **Highest Educational Attainment** |  |  |  |  |  |  |  |  |
| No Formal Education | Ref |  | Ref |  | Ref |  | Ref |  |
| Up to primary | 1.03 (0.78-1.37) | 0.825 | 0.90 (0.61-1.35) | 0.615 | 1.00 (-) |  | 0.00 (0.00-0.00) | <0.001 |
| Up to secondary | 1.61 (1.30-2.00) | <0.001 | 1.63 (1.24-2.14) | 0.001 | 0.15 (0.00-5.62) | 0.307 | 0.12 (0.08-0.18) | <0.001 |
| College and higher | 1.99 (1.45-2.72) | <0.001 | 2.33 (1.53-3.52) | <0.001 | 1.82 (0.10-32.81) | 0.686 | 0.33 (0.01-11.05) | 0.533 |
| **Marital Status** |  |  |  |  |  |  |  |  |
| Never married | Ref |  | Ref |  | Ref |  | Ref |  |
| Currently married | 0.62 (0.48-0.80) | <0.001 | 0.68 (0.51-0.90) | 0.009 | 0.12 (0.00-5.57) | 0.283 | 0.76 (0.33-1.74) | 0.505 |
| Divorced/widowed/separated | 0.64 (0.44-0.93) | 0.020 | 0.65 (0.44-0.94) | 0.025 | 0.18 (0.00-23.36) | 0.494 | 1.80 (0.31-10.29) | 0.506 |
| **Smoking Status** |  |  |  |  |  |  |  |  |
| Never Smoker | Ref |  | Ref |  | Ref |  | Ref |  |
| Current Smoker | 1.11 (0.78-1.57) | 0.556 | 0.95 (0.64-1.40) | 0.786 | 1.00 (-) |  | 0.00 (0.00-0.00) | <0.001 |
| Former Smoker | 1.01 (0.79-1.28) | 0.942 | 1.20 (0.87-1.66) | 0.252 | 2.53 (0.32-19.94) | 0.378 | 5.07 (2.25-11.43) | <0.001 |
| **Ever Alcohol Consumption** |  |  |  |  |  |  |  |  |
| Yes | Ref |  | Ref |  | Ref |  | Ref |  |
| No | 1.11 (0.92-1.35) | 0.259 | 1.08 (0.86-1.36) | 0.504 | 1.38 (0.19-9.96) | 0.752 | 1.45 (0.50-4.22) | 0.490 |
| **Teeth Cleaning Frequency** |  |  |  |  |  |  |  |  |
| Once a day | Ref |  | Ref |  | Ref |  | Ref |  |
| Twice a day | 1.42 (0.22-9.17) | 0.709 | 3.09 (0.44-21.84) | 0.256 | 1.75 (0.16-19.15) | 0.648 | 3.86 (0.35-42.11) | 0.264 |
| Infrequent/Never | 1.49 (0.15-15.09) | 0.734 | 1.13 (0.46-2.76) | 0.794 | 1.12 (0.08-16.13) | 0.933 | 1.36 (0.32-5.88) | 0.673 |

*AOR: Adjusted Odds Ratio; APR: Adjusted Prevalence Ratio; CI: Confidence Interval; COR: Crude Odds Ratio; CPR: Crude Prevalence Ratio*
