# Supplementary material for: Prevalence and associated factors of last dental visit and teeth cleaning frequency in Bangladesh, Bhutan, and Nepal: Findings from nationally representative surveys
Source: PLOS Glob Public Health. 2024 Jul 19;4(7):e0003511. doi: 10.1371/journal.pgph.0003511 (PMC11259307; doi:10.1371/journal.pgph.0003511)
Supplement: S11 Table — (DOCX) [file pgph.0003511.s011.docx]

**S11 Table: Crude and adjusted prevalence ratios and odds ratio for the factors associated with visiting a dentist in last six months in Nepal**

| **Characteristics** | **COR (95% CI)** | **P-value** | **CPR (95% CI)** | **P-value** | **AOR (95% CI)** | **P-value** | **APR (95% CI)** | **P-value** |
| --- | --- | --- | --- | --- | --- | --- | --- | --- |
| **Age Group (in years)** |  |  |  |  |  |  |  |  |
| 18–29 | Ref |  | Ref |  | Ref |  | Ref |  |
| 30-49 | 2.24 (1.25-4.02) | 0.007 | 2.44 (1.31-4.55) | 0.005 | 2.32 (1.22-4.43) | 0.01 | 2.34 (1.19-4.61) | 0.014 |
| 50-69 | 2.65 (1.43-4.89) | 0.002 | 2.65 (1.39-5.05) | 0.003 | 2.72 (1.29-5.71) | 0.008 | 2.40 (1.13-5.08) | 0.023 |
| **Gender** |  |  |  |  |  |  |  |  |
| Male | Ref |  | Ref |  | Ref |  | Ref |  |
| Female | 1.45 (0.96-2.19) | 0.076 | 2.17 (1.20-3.94) | 0.011 | 1.86 (1.14-3.04) | 0.013 | 3.12 (1.86-5.23) | <0.001 |
| **Highest Educational Attainment** |  |  |  |  |  |  |  |  |
| No Formal Education | Ref |  | Ref |  | Ref |  | Ref |  |
| Up to primary | 0.88 (0.56-1.37) | 0.574 | 0.81 (0.44-1.48) | 0.489 | 1.33 (0.81-2.19) | 0.255 | 1.52 (0.83-2.77) | 0.173 |
| Up to secondary | 0.72 (0.44-1.18) | 0.193 | 0.68 (0.37-1.26) | 0.218 | 1.42 (0.79-2.57) | 0.246 | 1.77 (0.83-3.79) | 0.141 |
| College and higher | 0.63 (0.18-2.13) | 0.454 | 0.79 (0.20-3.15) | 0.738 | 1.24 (0.34-4.54) | 0.75 | 2.35 (0.57-9.64) | 0.234 |
| **Marital Status** |  |  |  |  |  |  |  |  |
| Never married | Ref |  | Ref |  | Ref |  | Ref |  |
| Currently married | 1.71 (0.67-4.35) | 0.262 | 2.99 (1.08-8.30) | 0.036 | 0.94 (0.34-2.55) | 0.898 | 1.60 (0.52-4.89) | 0.408 |
| Divorced/widowed/separated | 2.14 (0.66-6.90) | 0.203 | 3.71 (0.92-14.92) | 0.065 | 0.90 (0.26-3.19) | 0.872 | 1.27 (0.27-5.85) | 0.762 |
| **Smoking Status** |  |  |  |  |  |  |  |  |
| Never Smoker | Ref |  | Ref |  | Ref |  | Ref |  |
| Current Smoker | 1.46 (0.93-2.28) | 0.101 | 1.88 (0.93-3.83) | 0.080 | 1.66 (0.98-2.81) | 0.058 | 3.24 (1.66-6.31) | 0.001 |
| Former Smoker | 2.42 (1.38-4.24) | 0.002 | 2.75 (1.28-5.90) | 0.010 | 2.60 (1.41-4.80) | 0.002 | 3.76 (1.75-8.10) | 0.001 |
| **Ever Alcohol Consumption** |  |  |  |  |  |  |  |  |
| Yes | Ref |  | Ref |  | Ref |  | Ref |  |
| No | 1.06 (0.69-1.61) | 0.791 | 1.19 (0.60-2.34) | 0.619 | 1.18 (0.73-1.93) | 0.497 | 1.46 (0.78-2.72) | 0.235 |
| **Teeth Cleaning Frequency** |  |  |  |  |  |  |  |  |
| Once a day | Ref |  | Ref |  | Ref |  | Ref |  |
| Twice a day | 1.23 (0.62-2.45) | 0.557 | 1.64 (0.66-4.06) | 0.284 | 1.28 (0.63-2.60) | 0.499 | 1.79 (0.73-4.39) | 0.201 |
| Infrequent/Never | 1.51 (0.87-2.60) | 0.140 | 1.56 (0.77-3.16) | 0.212 | 1.24 (0.70-2.19) | 0.467 | 1.17 (0.58-2.37) | 0.658 |

*AOR: Adjusted Odds Ratio; APR: Adjusted Prevalence Ratio; CI: Confidence Interval; COR: Crude Odds Ratio; CPR: Crude Prevalence Ratio*
