# Supplementary material for: Prevalence and associated factors of last dental visit and teeth cleaning frequency in Bangladesh, Bhutan, and Nepal: Findings from nationally representative surveys
Source: PLOS Glob Public Health. 2024 Jul 19;4(7):e0003511. doi: 10.1371/journal.pgph.0003511 (PMC11259307; doi:10.1371/journal.pgph.0003511)
Supplement: S12 Table — (DOCX) [file pgph.0003511.s012.docx]

**S12 Table: Crude and adjusted prevalence ratios and odds ratio for the factors associated with visiting a dentist in last twelve months in Bangladesh**

| **Characteristics** | **COR (95% CI)** | **P-value** | **CPR (95% CI)** | **P-value** | **AOR (95% CI)** | **P-value** | **APR (95% CI)** | **P-value** |
| --- | --- | --- | --- | --- | --- | --- | --- | --- |
| **Age Group (in years)** |  |  |  |  |  |  |  |  |
| 18–29 | Ref |  | Ref |  | Ref |  | Ref |  |
| 30-49 | 1.31 (1.11-1.55) | 0.001 | 1.25 (l.03-1.53) | 0.027 | 1.47 (1.23-1.77) | <0.001 | 1.36 (1.08-1.71) | 0.009 |
| 50-69 | 1.35 (1.11-1.64) | 0.003 | 1.35 (1.03-1.77) | 0.029 | 1.67 (1.34-2.10) | <0.001 | 1.61 (1.19-2.17) | 0.002 |
| **Gender** |  |  |  |  |  |  |  |  |
| Male | Ref |  | Ref |  | Ref |  | Ref |  |
| Female | 1.07 (0.94-1.21) | 0.312 | 1.06 (0.89-1.26) | 0.507 | 1.16 (0.97-1.41) | 0.117 | 1.12 (0.85-1.48) | 0.404 |
| **Highest Educational Attainment** |  |  |  |  |  |  |  |  |
| No Formal Education | Ref |  | Ref |  | Ref |  | Ref |  |
| Up to primary | 1.31 (1.12-1.53) | 0.001 | 1.20 (0.98-1.47) | 0.071 | 1.41 (1.20-1.67) | <0.001 | 1.37 (1.16-1.62) | <0.001 |
| Up to secondary | 1.27 (1.04-1.56) | 0.019 | 0.97 (0.72-1.31) | 0.851 | 1.42 (1.15-1.76) | 0.001 | 1.16 (0.87-1.55) | 0.324 |
| College and higher | 1.65 (1.27-2.14) | <0.001 | 1.37 (0.97-1.94) | 0.077 | 1.78 (1.36-2.33) | <0.001 | 1.59 (1.11-2.26) | 0.011 |
| **Marital Status** |  |  |  |  |  |  |  |  |
| Never married | Ref |  | Ref |  | Ref |  | Ref |  |
| Currently married | 1.07 (0.82-1.39) | 0.619 | 1.11 (0.83-1.50) | 0.480 | 0.84 (0.62-1.14) | 0.275 | 0.89 (0.62-1.29) | 0.543 |
| Divorced/widowed/separated | 0.99 (0.67-1.47) | 0.970 | 1.09 (0.63-1.87) | 0.766 | 0.73 (0.47-1.14) | 0.170 | 0.77 (0.42-1.41) | 0.395 |
| **Smoking Status** |  |  |  |  |  |  |  |  |
| Never Smoker | Ref |  | Ref |  | Ref |  | Ref |  |
| Current Smoker | 0.87 (0.74-1.02) | 0.089 | 0.92 (0.73-1.15) | 0.444 | 0.93 (0.75-1.15) | 0.504 | 0.96 (0.70-1.32) | 0.819 |
| Former Smoker | 1.19 (0.94-1.49) | 0.151 | 1.21 (0.91-1.61) | 0.192 | 1.20 (0.92-1.58) | 0.183 | 1.20 (0.85-1.70) | 0.298 |
| **Ever Alcohol Consumption** |  |  |  |  |  |  |  |  |
| Yes | Ref |  | Ref |  | Ref |  | Ref |  |
| No | 0.93 (0.73-1.17) | 0.528 | 1.14 (0.83-1.57) | 0.409 | 0.85 (0.66-1.10) | 0.219 | 1.06 (0.77-1.46) | 0.720 |
| **Teeth Cleaning Frequency** |  |  |  |  |  |  |  |  |
| Once a day | Ref |  | Ref |  | Ref |  | Ref |  |
| Twice a day | 1.09 (0.96-1.24) | 0.200 | 0.96 (0.80-1.14) | 0.611 | 1.03 (0.90-1.18) | 0.657 | 0.91 (0.76-1.10) | 0.339 |
| Infrequent/Never | 0.84 (0.37-1.91) | 0.684 | 0.41 (0.16-1.09) | 0.073 | 0.89 (0.39-2.03) | 0.787 | 0.40 (0.15-1.04) | 0.061 |

*AOR: Adjusted Odds Ratio; APR: Adjusted Prevalence Ratio; CI: Confidence Interval; COR: Crude Odds Ratio; CPR: Crude Prevalence Ratio*
