# Supplementary material for: Prevalence and associated factors of last dental visit and teeth cleaning frequency in Bangladesh, Bhutan, and Nepal: Findings from nationally representative surveys
Source: PLOS Glob Public Health. 2024 Jul 19;4(7):e0003511. doi: 10.1371/journal.pgph.0003511 (PMC11259307; doi:10.1371/journal.pgph.0003511)
Supplement: S13 Table — (DOCX) [file pgph.0003511.s013.docx]

**S13 Table: Crude and adjusted prevalence ratios and odds ratio for the factors associated with visiting a dentist in last twelve months in Bhutan**

| **Characteristics** | **COR (95% CI)** | **P-value** | **CPR (95% CI)** | **P-value** | **AOR (95% CI)** | **P-value** | **APR (95% CI)** | **P-value** |
| --- | --- | --- | --- | --- | --- | --- | --- | --- |
| **Age Group (in years)** |  |  |  |  |  |  |  |  |
| 18–29 | Ref |  | Ref |  | Ref |  | Ref |  |
| 30-49 | 0.74 (0.62-0.89) | 0.024 | 0.80 (0.66-0.97) | 0.024 | 0.51 (0.11-2.34) | 0.389 | 0.36 (0.10-1.33) | 0.009 |
| 50-69 | 0.72 (0.58-0.90) | 0.022 | 0.77 (0.62-0.96) | 0.022 | 1.00 (-) |  | 0.00 (0.00-0.00) | 0.002 |
| **Gender** |  |  |  |  |  |  |  |  |
| Male | Ref |  | Ref |  | Ref |  | Ref |  |
| Female | 1.14 (0.98-1.34) | 0.072 | 1.17 (0.99-1.40) | 0.072 | 0.61 (0.13-2.74) | 0.516 | 0.63 (0.11-3.43) | 0.404 |
| **Highest Educational Attainment** |  |  |  |  |  |  |  |  |
| No Formal Education | Ref |  | Ref |  | Ref |  | Ref |  |
| Up to primary | 1.05 (0.83-1.33) | 0.418 | 0.89 (0.66-1.19) | 0.418 | 1.00 (-) |  | 0.00 (0.00-0.00) | <0.001 |
| Up to secondary | 1.61 (1.34-1.93) | <0.001 | 1.53 (1.26-1.86) | <0.001 | 0.55 (0.08-3.97) | 0.554 | 0.44 (0.13-1.47) | 0.324 |
| College and higher | 2.26 (1.73-2.95) | <0.001 | 2.32 (1.75-3.08) | <0.001 | 0.88 (0.06-13.44) | 0.928 | 0.09 (0.01-1.42) | 0.011 |
| **Marital Status** |  |  |  |  |  |  |  |  |
| Never married | Ref |  | Ref |  | Ref |  | Ref |  |
| Currently married | 0.77 (0.62-0.97) | 0.066 | 0.79 (0.62-1.02) | 0.066 | 0.26 (0.02-2.80) | 0.269 | 3.38 (0.54-21.04) | 0.543 |
| Divorced/widowed/separated | 0.73 (0.52-1.01) | 0.019 | 0.68 (0.50-0.94) | 0.019 | 0.15 (0.00-6.92) | 0.332 | 2.53 (1.36-4.70) | 0.395 |
| **Smoking Status** |  |  |  |  |  |  |  |  |
| Never Smoker | Ref |  | Ref |  | Ref |  | Ref |  |
| Current Smoker | 1.04 (0.77-1.41) | 0.395 | 0.87 (0.64-1.19) | 0.395 | 1.00 (-) |  | 0.00 (0.00-0.00) | 0.819 |
| Former Smoker | 1.00 (0.82-1.22) | 0.263 | 1.16 (0.89-1.52) | 0.263 | 1.46 (0.28-7.50) | 0.65 | 2.41 (0.39-15.01) | 0.298 |
| **Ever Alcohol Consumption** |  |  |  |  |  |  |  |  |
| Yes | Ref |  | Ref |  | Ref |  | Ref |  |
| No | 1.10 (0.94-1.29) | 0.603 | 1.06 (0.86-1.30) | 0.603 | 0.60 (0.12-2.92) | 0.529 | 0.28 (0.04-1.81) | 0.720 |
| **Teeth Cleaning Frequency** |  |  |  |  |  |  |  |  |
| Once a day | Ref |  | Ref |  | Ref |  | Ref |  |
| Twice a day | 2.10 (0.51-8.67) | 0.038 | 4.48 (1.09-18.46) | 0.038 | 2.64 (0.42-16.67) | 0.303 | 6.44 (1.63-25.47) | 0.339 |
| Infrequent/Never | 0.88 (0.10-7.96) | 0.187 | 0.33 (0.06-1.74) | 0.187 | 1.37 (0.12-15.68) | 0.801 | 0.30 (0.02-4.02) | 0.061 |

*AOR: Adjusted Odds Ratio; APR: Adjusted Prevalence Ratio; CI: Confidence Interval; COR: Crude Odds Ratio; CPR: Crude Prevalence Ratio*
