# Supplementary material for: Prevalence and associated factors of last dental visit and teeth cleaning frequency in Bangladesh, Bhutan, and Nepal: Findings from nationally representative surveys
Source: PLOS Glob Public Health. 2024 Jul 19;4(7):e0003511. doi: 10.1371/journal.pgph.0003511 (PMC11259307; doi:10.1371/journal.pgph.0003511)
Supplement: S14 Table — (DOCX) [file pgph.0003511.s014.docx]

**S14 Table: Crude and adjusted prevalence ratios and odds ratio for the factors associated with visiting a dentist in last twelve months in Nepal**

| **Characteristics** | **COR (95% CI)** | **P-value** | **CPR (95% CI)** | **P-value** | **AOR (95% CI)** | **P-value** | **APR (95% CI)** | **P-value** |
| --- | --- | --- | --- | --- | --- | --- | --- | --- |
| **Age Group (in years)** |  |  |  |  |  |  |  |  |
| 18–29 | Ref |  | Ref |  | Ref |  | Ref |  |
| 30-49 | 1.86 (1.21-2.85) | 0.004 | 1.79 (1.14-2.80) | 0.012 | 1.98 (1.23-3.19) | 0.005 | 1.83 (1.11-3.02) | 0.018 |
| 50-69 | 2.31 (1.47-3.62) | <0.001 | 2.35 (1.37-4.03) | 0.002 | 2.54 (1.46-4.42) | 0.001 | 2.49 (1.42-4.36) | 0.001 |
| **Gender** |  |  |  |  |  |  |  |  |
| Male | Ref |  | Ref |  | Ref |  | Ref |  |
| Female | 1.56 (1.12-2.16) | 0.008 | 2.44 (1.44-4.12) | 0.001 | 1.93 (1.30-2.86) | 0.001 | 3.87 (2.07-7.24) | <0.001 |
| **Highest Educational Attainment** |  |  |  |  |  |  |  |  |
| No Formal Education | Ref |  | Ref |  | Ref |  | Ref |  |
| Up to primary | 0.66 (0.46-0.95) | 0.026 | 0.65 (0.41-1.04) | 0.073 | 0.96 (0.64-1.44) | 0.852 | 1.21 (0.78-1.88) | 0.402 |
| Up to secondary | 0.73 (0.50-1.07) | 0.103 | 0.77 (0.48-1.25) | 0.296 | 1.34 (0.84-2.12) | 0.219 | 1.99 (1.17-3.39) | 0.012 |
| College and higher | 0.69 (0.29-1.63) | 0.396 | 0.68 (0.24-1.93) | 0.467 | 1.25 (0.50-3.11) | 0.635 | 2.00 (0.66-6.02) | 0.216 |
| **Marital Status** |  |  |  |  |  |  |  |  |
| Never married | Ref |  | Ref |  | Ref |  | Ref |  |
| Currently married | 1.58 (0.78-3.21) | 0.209 | 2.44 (0.91-6.53) | 0.075 | 0.96 (0.45-2.06) | 0.917 | 1.48 (0.52-4.21) | 0.466 |
| Divorced/widowed/separated | 1.40 (0.55-3.59) | 0.481 | 2.50 (0.76-8.22) | 0.130 | 0.61 (0.22-1.69) | 0.346 | 0.88 (0.25-3.15) | 0.845 |
| **Smoking Status** |  |  |  |  |  |  |  |  |
| Never Smoker | Ref |  | Ref |  | Ref |  | Ref |  |
| Current Smoker | 1.34 (0.94-1.91) | 0.102 | 1.76 (1.08-2.86) | 0.022 | 1.55 (1.02-2.35) | 0.038 | 2.75 (1.68-4.50) | <0.001 |
| Former Smoker | 1.94 (1.23-3.07) | 0.005 | 2.27 (1.19-4.33) | 0.013 | 2.10 (1.28-3.46) | 0.004 | 2.76 (1.42-5.36) | 0.003 |
| **Ever Alcohol Consumption** |  |  |  |  |  |  |  |  |
| Yes | Ref |  | Ref |  | Ref |  | Ref |  |
| No | 1.14 (0.82-1.60) | 0.439 | 0.97 (0.62-1.50) | 0.877 | 1.15 (0.77-1.70) | 0.495 | 0.96 (0.56-1.65) | 0.877 |
| **Teeth Cleaning Frequency** |  |  |  |  |  |  |  |  |
| Once a day | Ref |  | Ref |  | Ref |  | Ref |  |
| Twice a day | 1.53 (0.89-2.62) | 0.121 | 1.46 (0.72-2.95) | 0.295 | 1.54 (0.88-2.68) | 0.127 | 1.49 (0.74-2.98) | 0.261 |
| Infrequent/Never | 1.49 (0.95-2.34) | 0.081 | 1.43 (0.75-2.72) | 0.272 | 1.22 (0.76-1.97) | 0.406 | 1.06 (0.55-2.02) | 0.865 |

*AOR: Adjusted Odds Ratio; APR: Adjusted Prevalence Ratio; CI: Confidence Interval; COR: Crude Odds Ratio; CPR: Crude Prevalence Ratio*
