# Supplementary material for: Prevalence and associated factors of last dental visit and teeth cleaning frequency in Bangladesh, Bhutan, and Nepal: Findings from nationally representative surveys
Source: PLOS Glob Public Health. 2024 Jul 19;4(7):e0003511. doi: 10.1371/journal.pgph.0003511 (PMC11259307; doi:10.1371/journal.pgph.0003511)
Supplement: S15 Table — (DOCX) [file pgph.0003511.s015.docx]

**S15 Table: Crude and adjusted prevalence ratios and odds ratio for the factors associated with never visiting a dentist in Bangladesh**

| **Characteristics** | **COR (95% CI)** | **P-value** | **CPR (95% CI)** | **P-value** | **AOR (95% CI)** | **P-value** | **APR (95% CI)** | **P-value** |
| --- | --- | --- | --- | --- | --- | --- | --- | --- |
| **Age Group (in years)** |  |  |  |  |  |  |  |  |
| 18–29 | Ref |  | Ref |  | Ref |  | Ref |  |
| 30-49 | 0.61 (0.54-0.70) | <0.001 | 0.87 (0.83-0.91) | 0.840 | 0.55 (0.48-0.63) | <0.001 | 0.84 (0.80-0.88) | <0.001 |
| 50-69 | 0.53 (0.45-0.61) | <0.001 | 0.82 (0.76-0.87) | 0.771 | 0.42 (0.36-0.51) | <0.001 | 0.77 (0.72-0.83) | <0.001 |
| **Gender** |  |  |  |  |  |  |  |  |
| Male | Ref |  | Ref |  | Ref |  | Ref |  |
| Female | 0.96 (0.87-1.06) | 0.454 | 1.00 (0.96-1.04) | 1.011 | 0.90 (0.78-1.04) | 0.159 | 1.01 (0.95-1.07) | 0.721 |
| **Highest Educational Attainment** |  |  |  |  |  |  |  |  |
| No Formal Education | Ref |  | Ref |  | Ref |  | Ref |  |
| Up to primary | 0.78 (0.69-0.88) | <0.001 | 0.96 (0.92-1.01) | 0.879 | 0.66 (0.58-0.75) | <0.001 | 0.88 (0.84-0.92) | <0.001 |
| Up to secondary | 0.75 (0.64-0.87) | <0.001 | 1.00 (0.93-1.07) | 0.879 | 0.58 (0.49-0.68) | <0.001 | 0.88 (0.82-0.94) | <0.001 |
| College and higher | 0.47 (0.38-0.58) | <0.001 | 0.84 (0.75-0.95) | 0.765 | 0.38 (0.31-0.48) | <0.001 | 0.77 (0.68-0.87) | <0.001 |
| **Marital Status** |  |  |  |  |  |  |  |  |
| Never married | Ref |  | Ref |  | Ref |  | Ref |  |
| Currently married | 0.73 (0.59-0.90) | 0.004 | 0.91 (0.85-0.97) | 0.970 | 0.99 (0.78-1.26) | 0.958 | 0.97 (0.90-1.04) | 0.406 |
| Divorced/widowed/separated | 0.59 (0.44-0.80) | 0.001 | 0.82 (0.70-0.96) | 0.923 | 0.88 (0.63-1.25) | 0.483 | 0.92 (0.78-1.09) | 0.332 |
| **Smoking Status** |  |  |  |  |  |  |  |  |
| Never Smoker | Ref |  | Ref |  | Ref |  | Ref |  |
| Current Smoker | 1.11 (0.99-1.25) | 0.083 | 1.02 (0.97-1.08) | 1.051 | 1.10 (0.93-1.30) | 0.268 | 1.05 (0.98-1.13) | 0.172 |
| Fomer Smoker | 0.88 (0.73-1.05) | 0.162 | 0.92 (0.84-1.01) | 0.987 | 0.96 (0.78-1.19) | 0.727 | 0.99 (0.89-1.10) | 0.801 |
| **Ever Alcohol Consumption** |  |  |  |  |  |  |  |  |
| Yes | Ref |  | Ref |  | Ref |  | Ref |  |
| No | 1.12 (0.93-1.34) | 0.244 | 0.98 (0.90-1.07) | 1.029 | 1.24 (1.01-1.52) | 0.036 | 1.03 (0.94-1.13) | 0.539 |
| **Teeth Cleaning Frequency** |  |  |  |  |  |  |  |  |
| Once a day | Ref |  | Ref |  | Ref |  | Ref |  |
| Twice a day | 0.88 (0.80-0.98) | 0.015 | 0.98 (0.94-1.03) | 1.002 | 1.03 (0.90-1.18) | 0.657 | 1.00 (0.96-1.05) | 0.927 |
| Infrequent/Never | 0.87 (0.49-1.55) | 0.641 | 0.95 (0.74-1.21) | 0.965 | 0.89 (0.39-2.03) | 0.787 | 0.96 (0.76-1.23) | 0.773 |

*AOR: Adjusted Odds Ratio; APR: Adjusted Prevalence Ratio; CI: Confidence Interval; COR: Crude Odds Ratio; CPR: Crude Prevalence Ratio*
