# Supplementary material for: Prevalence and associated factors of last dental visit and teeth cleaning frequency in Bangladesh, Bhutan, and Nepal: Findings from nationally representative surveys
Source: PLOS Glob Public Health. 2024 Jul 19;4(7):e0003511. doi: 10.1371/journal.pgph.0003511 (PMC11259307; doi:10.1371/journal.pgph.0003511)
Supplement: S16 Table — (DOCX) [file pgph.0003511.s016.docx]

**S16 Table: Crude and adjusted prevalence ratios and odds ratio for the factors associated with never visiting a dentist in Bhutan**

| **Characteristics** | **COR (95% CI)** | **P-value** | **CPR (95% CI)** | **P-value** | **AOR (95% CI)** | **P-value** | **APR (95% CI)** | **P-value** |
| --- | --- | --- | --- | --- | --- | --- | --- | --- |
| **Age Group (in years)** |  |  |  |  |  |  |  |  |
| 18–29 | Ref |  | Ref |  | Ref |  | Ref |  |
| 30-49 | 1.23 (1.07-1.41) | 0.004 | 1.10 (1.01-1.20) | 0.038 | 0.73 (0.23-2.35) | 0.597 | 1.01 (0.01-0.63) | 0.532 |
| 50-69 | 0.91 (0.77-1.07) | 0.258 | 0.97 (0.87-1.09) | 0.617 | 1.57 (0.33-7.41) | 0.567 | 1.01 (0.01-1.22) | 0.227 |
| **Gender** |  |  |  |  |  |  |  |  |
| Male | Ref |  | Ref |  | Ref |  | Ref |  |
| Female | 0.85 (0.76-0.96) | 0.007 | 0.94 (0.88-1.01) | 0.112 | 2.03 (0.77-5.31) | 0.151 | 1.01 (0.01-0.99) | 0.326 |
| **Highest Educational Attainment** |  |  |  |  |  |  |  |  |
| No Formal Education | Ref |  | Ref |  | Ref |  | Ref |  |
| Up to primary | 0.99 (0.84-1.16) | 0.885 | 0.96 (0.87-1.05) | 0.331 | 1.17 (0.24-5.66) | 0.844 | 1.01 (0.01-1.58) | 0.119 |
| Up to secondary | 0.64 (0.55-0.73) | <0.001 | 0.75 (0.68-0.83) | <0.001 | 1.56 (0.39-6.28) | 0.531 | 1.01 (0.01-1.26) | 0.211 |
| College and higher | 0.45 (0.35-0.57) | <0.001 | 0.57 (0.47-0.68) | <0.001 | 0.54 (0.07-4.11) | 0.549 | 1.02 (0.01-1.34) | 0.183 |
| **Marital Status** |  |  |  |  |  |  |  |  |
| Never married | Ref |  | Ref |  | Ref |  | Ref |  |
| Currently married | 1.25 (1.05-1.50) | 0.014 | 1.18 (1.02-1.36) | 0.027 | 1.05 (0.20-5.50) | 0.953 | 0.99 (0.01--0.61) | 0.541 |
| Divorced/widowed/separated | 1.21 (0.95-1.54) | 0.130 | 1.11 (0.93-1.33) | 0.231 | 3.40 (0.19-60.14) | 0.403 | 1.00 (0.01--0.20) | 0.845 |
| **Smoking Status** |  |  |  |  |  |  |  |  |
| Never Smoker | Ref |  | Ref |  | Ref |  | Ref |  |
| Current Smoker | 1.15 (0.91-1.45) | 0.235 | 1.03 (0.90-1.18) | 0.701 | 1.00 (-) |  | 1.01 (0.01-1.34) | 0.184 |
| Former Smoker | 0.92 (0.79-1.07) | 0.292 | 0.91 (0.83-1.01) | 0.090 | 1.37 (0.38-4.91) | 0.625 | 1.00 (0.01-0.24) | 0.808 |
| **Ever Alcohol Consumption** |  |  |  |  |  |  |  |  |
| Yes | Ref |  | Ref |  | Ref |  | Ref |  |
| No | 1.03 (0.91-1.16) | 0.677 | 1.06 (0.98-1.15) | 0.152 | 1.21 (0.46-3.18) | 0.704 | 1.00 (0.00-0.42) | 0.679 |
| **Teeth Cleaning Frequency** |  |  |  |  |  |  |  |  |
| Once a day | Ref |  | Ref |  | Ref |  | Ref |  |
| Twice a day | 0.87 (0.30-2.48) | 0.790 | 0.99 (0.96-1.01) | 0.328 | 0.92 (0.28-2.96) | 0.886 | 0.99 (0.01-1.08) | 0.282 |
| Infrequent/Never | 0.61 (0.19-1.98) | 0.409 | 1.00 (0.99-1.00) | 0.343 | 0.63 (0.19-2.16) | 0.465 | 1.00 (0.00-0.79) | 0.432 |

*AOR: Adjusted Odds Ratio; APR: Adjusted Prevalence Ratio; CI: Confidence Interval; COR: Crude Odds Ratio; CPR: Crude Prevalence Ratio*
