# Supplementary material for: Prevalence and associated factors of last dental visit and teeth cleaning frequency in Bangladesh, Bhutan, and Nepal: Findings from nationally representative surveys
Source: PLOS Glob Public Health. 2024 Jul 19;4(7):e0003511. doi: 10.1371/journal.pgph.0003511 (PMC11259307; doi:10.1371/journal.pgph.0003511)
Supplement: S17 Table — (DOCX) [file pgph.0003511.s017.docx]

**S17 Table: Crude and adjusted prevalence ratios and odds ratio for the factors associated with never visiting a dentist in Nepal**

| **Characteristics** | **COR (95% CI)** | **P-value** | **CPR (95% CI)** | **P-value** | **AOR (95% CI)** | **P-value** | **APR (95% CI)** | **P-value** |
| --- | --- | --- | --- | --- | --- | --- | --- | --- |
| **Age Group (in years)** |  |  |  |  |  |  |  |  |
| 18–29 | Ref |  | Ref |  | Ref |  | Ref |  |
| 30-49 | 0.45 (0.33-0.62) | <0.001 | 0.96 (0.95-0.98) | <0.001 | 0.41 (0.29-0.59) | <0.001 | 0.97 (0.95-0.98) | <0.001 |
| 50-69 | 0.36 (0.26-0.51) | <0.001 | 0.94 (0.92-0.97) | <0.001 | 0.32 (0.21-0.48) | <0.001 | 0.95 (0.92-0.98) | <0.001 |
| **Gender** |  |  |  |  |  |  |  |  |
| Male | Ref |  | Ref |  | Ref |  | Ref |  |
| Female | 0.60 (0.48-0.77) | <0.001 | 0.96 (0.95-0.98) | <0.001 | 0.50 (0.37-0.67) | <0.001 | 0.95 (0.93-0.97) | <0.001 |
| **Highest Educational Attainment** |  |  |  |  |  |  |  |  |
| No Formal Education | Ref |  | Ref |  | Ref |  | Ref |  |
| Up to primary | 1.32 (1.02-1.72) | 0.036 | 1.03 (1.01-1.05) | 0.015 | 0.84 (0.63-1.13) | 0.248 | 1.01 (0.98-1.02) | 0.934 |
| Up to secondary | 1.50 (1.14-1.98) | 0.004 | 1.03 (1.01-1.06) | 0.008 | 0.73 (0.52-1.03) | 0.077 | 1.01 (0.97-1.02) | 0.481 |
| College and higher | 1.78 (0.95-3.34) | 0.073 | 1.04 (1.00-1.08) | 0.054 | 0.86 (0.44-1.69) | 0.661 | 1.00 (0.95-1.03) | 0.680 |
| **Marital Status** |  |  |  |  |  |  |  |  |
| Never married | Ref |  | Ref |  | Ref |  | Ref |  |
| Currently married | 0.57 (0.34-0.96) | 0.036 | 0.96 (0.94-0.98) | <0.001 | 1.02 (0.58-1.79) | 0.953 | 0.94 (0.97-1.02) | 0.633 |
| Divorced/widowed/separated | 0.44 (0.23-0.84) | 0.014 | 0.93 (0.88-0.99) | 0.031 | 1.09 (0.53-2.24) | 0.816 | 0.88 (0.93-1.07) | 0.987 |
| **Smoking Status** |  |  |  |  |  |  |  |  |
| Never Smoker | Ref |  | Ref |  | Ref |  | Ref |  |
| Current Smoker | 0.78 (0.60-1.02) | 0.066 | 0.97 (0.95-1.00) | 0.022 | 0.69 (0.50-0.93) | 0.016 | 0.95 (0.93-0.98) | 0.001 |
| Former Smoker | 0.58 (0.41-0.82) | 0.002 | 0.92 (0.87-0.97) | 0.004 | 0.55 (0.38-0.80) | 0.002 | 0.87 (0.97-0.97) | 0.003 |
| **Ever Alcohol Consumption** |  |  |  |  |  |  |  |  |
| Yes | Ref |  | Ref |  | Ref |  | Ref |  |
| No | 0.79 (0.62-1.02) | 0.067 | 1.00 (0.98-1.02) | 0.869 | 0.82 (0.61-1.09) | 0.176 | 0.98 (0.97-1.02) | 0.674 |
| **Teeth Cleaning Frequency** |  |  |  |  |  |  |  |  |
| Once a day | Ref |  | Ref |  | Ref |  | Ref |  |
| Twice a day | 0.73 (0.48-1.10) | 0.137 | 0.99 (0.95-1.03) | 0.581 | 0.70 (0.46-1.07) | 0.102 | 0.95 (0.95-1.02) | 0.392 |
| Infrequent/Never | 0.68 (0.49-0.94) | 0.021 | 0.98 (0.95-1.01) | 0.265 | 0.83 (0.59-1.18) | 0.293 | 0.95 (0.97-1.03) | 0.982 |

*AOR: Adjusted Odds Ratio; APR: Adjusted Prevalence Ratio; CI: Confidence Interval; COR: Crude Odds Ratio; CPR: Crude Prevalence Ratio*
