# Supplementary material for: Prevalence and associated factors of last dental visit and teeth cleaning frequency in Bangladesh, Bhutan, and Nepal: Findings from nationally representative surveys
Source: PLOS Glob Public Health. 2024 Jul 19;4(7):e0003511. doi: 10.1371/journal.pgph.0003511 (PMC11259307; doi:10.1371/journal.pgph.0003511)
Supplement: Attachment — Submitted filename: Response to Reviewer.docx [file pgph.0003511.s018.docx]

**Ref No:** PGPH-D-24-00051

**Submitted Title:** Dental service utilization and oral hygiene practice in Bangladesh, Bhutan, and Nepal: Findings from nationally representative surveys

**Revised Title:** Prevalence and associated factors of last dental visit and teeth cleaning frequency in Bangladesh, Bhutan, and Nepal: Findings from nationally representative surveys

**Response to reviewers’ comments:**

**Reviewer 1:**

*interesting topic.*

*well designed paper*

*Good English language*

*Adequate tables.*

*The introduction and Discussion sections should be implemented with the topic about the importance of oral hygiene from the pregnancy and in pre-school period (please cite PMID: 29178747; PMID: 28462177; PMID: 30789195; PMID: 31110611*

**Response:** Thank you for the valuable comment! We have revised the introduction and discussion section about the importance of oral hygiene during the pregnancy and in the pre-school period. We have also cited the mentioned articles. Please see page no. 4: “Evidence suggests that oral diseases, especially periodontitis, are associated with systemic conditions, including cardiovascular disease, diabetes mellitus, and adverse pregnancy outcomes such as low birth weight [9–12] .” and page no. 19 : “For children in the preschool period, early oral hygiene practices are essential for preventing dental caries, the most common chronic disease in childhood. Good oral hygiene habits, including regular brushing and dental check-ups, can prevent tooth decay and its associated pain and complications, affecting eating, speaking, and overall quality of life [46,47]. Moreover, early dental visits help inculcate a positive attitude towards dental health, laying the foundation for healthy oral hygiene practices that can last a lifetime [48]. Previous studies also showed that poor knowledge and awareness of parents and caregivers about potential risk factors of oral health are associated with infant and child oral health risk status [49,50]. There is a need for school-based oral health prevention programs, emphasizing the blend of theoretical knowledge and practical application for effective learning [51,52].”

**Reviewer 2:**

*In view that you assessed only one oral hygiene practice (toothbrushing) and one dental service utilization variable (last visit to the dentist), the title is too widely worded hence misleading.*

**Response:** Based on the suggestion, the authors have revised the title: “Prevalence and associated factors of last dental visit and teeth cleaning frequency in Bangladesh, Bhutan, and Nepal: Findings from nationally representative surveys”.

*Since the fundamental reason for toothbrushing and dental visits are dental caries and periodontitis, some numerical data re. prevalence of caries and periodontitis in the 3 countries is relevant in the introduction to put things in proper perspective.*

**Response:** Thank you for the insightful suggestion! In the revised manuscript, we have included the prevalence data about dental caries and periodontitis among the residents of all three countries in the introduction section: “Data from the World Health Organization (WHO) indicates that in 2022, the rates of untreated caries in permanent teeth for individuals aged 5 and older were 30.4% in Bangladesh, 29.7% in Bhutan, and 31.5% in Nepal [20–22]. Concurrently, the occurrence of severe periodontal disease among those aged 15 and above was recorded at 23.4% in Bangladesh, 14.8% in Bhutan, and 23.5% in Nepal during the same year [20–22].”

*References are not pin-pointed. For example Ref 17 is several times used with no page numbers. It is very difficult for the reader to widen his view of the issue from the reference this way. It is not feasable to read the whole refereed book, or even article which is many pages long, like ref 16.*

**Response:** Thank you for your feedback regarding the references. Based on your suggestion, we have carefully reviewed the reference section to ensure adherence to the Vancouver referencing system, in accordance with the submission guidelines of the PLOS Global Public Health journal, available here: <https://journals.plos.org/globalpublichealth/s/submission-guidelines#loc-reference-style> .

As the journal’s referencing style does not require specifying specific pages within a citation, page numbers have been included for references where available. Additionally, we have supplemented any missing information in the references.
