# Supplementary material for: Prevalence and associated factors of last dental visit and teeth cleaning frequency in Bangladesh, Bhutan, and Nepal: Findings from nationally representative surveys
Source: PLOS Glob Public Health. 2024 Jul 19;4(7):e0003511. doi: 10.1371/journal.pgph.0003511 (PMC11259307; doi:10.1371/journal.pgph.0003511)
Supplement: Attachment — Submitted filename: Plos review_RBR.docx [file pgph.0003511.s019.docx]

1. The methodology mentions data collection through questionnaire interviews as well as physical and biochemical measurements conducted by trained personnel. However, it does not provide detailed information on the primary data collection methods, which could include specifics on the questionnaire design, training of personnel, and quality control measures. This lack of detail might make it difficult to assess the robustness of the primary data.

2. While results based on secondary data may have limitations compared to primary data due to factors like data quality and availability of variables, authors can still provide valuable insights, especially when analysing large-scale datasets like those from the WHO. However, it's essential to acknowledge and consider the limitations inherent in secondary data analysis.

3. The methodology doesn't explicitly mention whether the data from each country is proportional to its diverse population. It's crucial to ensure that the sample adequately represents the population of interest to generalise the findings accurately.

4. The methodology mentions including various sociodemographic factors as independent variables in the analysis. I would opine that it is essential to explicitly clarify whether any specific adjustment methods (e.g., regression modelling) were used to account for these variables' potential confounding effects on the outcome variables.

5. Confounders can distort the true relationship between the independent and dependent variables. The methodology mentions a Directed Acyclic Graph (DAG) showing potential confounding paths between the variables. However, it doesn't explicitly state whether all identified confounders were accounted for in the analysis. The authors could clarify which confounders were considered and how they were addressed in the analysis.

6. Standardisation methods can help control for confounding variables in statistical analysis. The methodology doesn't mention specific standardization techniques used to neutralize confounders. The authors need to explicitly clarify if any standardisation or adjustment methods were employed to mitigate confounding effects.

8. The background information provided by the authors suggests that adherence to oral hygiene practices has been linked to higher socioeconomic status and education level. However, the specific context of Bangladesh, Bhutan, and Nepal may differ from previous studies, and the authors could consider mentioning that the aim was also to investigate the associations between oral health practices and sociodemographic factors within these countries.
