# Supplementary material for: Prevalence and associated factors of last dental visit and teeth cleaning frequency in Bangladesh, Bhutan, and Nepal: Findings from nationally representative surveys
Source: PLOS Glob Public Health. 2024 Jul 19;4(7):e0003511. doi: 10.1371/journal.pgph.0003511 (PMC11259307; doi:10.1371/journal.pgph.0003511)
Supplement: Attachment — Submitted filename: Response to Reviewer.docx [file pgph.0003511.s020.docx]

**Submitted Title:** Prevalence and associated factors of last dental visit and teeth cleaning frequency in Bangladesh, Bhutan, and Nepal: Findings from nationally representative surveys

**Ref:** PGPH-D-24-00051R1

*1. The methodology mentions data collection through questionnaire interviews as well as physical and biochemical measurements conducted by trained personnel. However, it does not provide detailed information on the primary data collection methods, which could include specifics on the questionnaire design, training of personnel, and quality control measures. This lack of detail might make it difficult to assess the robustness of the primary data.*

**Response:** Thanks! I have revised the data collection section as following: “Data collection across three countries employed the WHO NCD STEPS instrument, incorporating three steps with core, expanded, and country-specific questions to measure NCD risk factors. In Bangladesh, the survey included essential and selected optional modules such as oral health and cervical cancer screening and was translated into the local language with subsequent validation through back translation [18,27,28]. Trained enumerators and supervisors, who had undergone extensive training on interviewing techniques, household selection, and data recording on Android tablets also received practical training on physical and biochemical measurements. Mock interviews were conducted in local dialects to ensure the quality and standardization of the process. Supervisors specifically focused on overseeing the data collection, ensuring data accuracy, and providing necessary support [18,27,28].

During the survey, participants were interviewed face-to-face to collect sociodemographic information, behavioral risk factors like smoking and alcohol use, and oral hygiene practices. Upon giving written consent, each participant completed STEP I and II questionnaires received a QR-coded urine container and a feedback form outlining their health metrics. Additionally, participants were given an appointment card detailing fasting instructions for subsequent biochemical measurements involving blood glucose and lipid assessments. The entire data collection process was facilitated using Android tablets with ODK software, and data were uploaded to a cloud-based server for analysis [18,27,28].”

*2. While results based on secondary data may have limitations compared to primary data due to factors like data quality and availability of variables, authors can still provide valuable insights, especially when analysing large-scale datasets like those from the WHO. However, it's essential to acknowledge and consider the limitations inherent in secondary data analysis.*

**Response:** Thank you for this important comment. We have added the following as in the limitation section: “This study utilized secondary datasets, which provide valuable insights through extensive coverage and standardized methodologies but is limited by data quality variability and the potential misalignment of variables with our specific research objectives.”

*3. The methodology doesn't explicitly mention whether the data from each country is proportional to its diverse population. It's crucial to ensure that the sample adequately represents the population of interest to generalise the findings accurately.*

**Response:** Thanks! We have already mentioned in under the ‘MATERIALS AND METHODS’ section and the study design, setting and sampling subsection: “A secondary analysis was conducted on the WHO STEPwise approach to surveillance (STEPS) surveys from Bangladesh, Bhutan, and Nepal. These nationally representative STEPS surveys aimed to update health indicators for select non-communicable diseases in the respective countries.” WHO STEPs data are nationally representative.

*4. The methodology mentions including various sociodemographic factors as independent variables in the analysis. I would opine that it is essential to explicitly clarify whether any specific adjustment methods (e.g., regression modelling) were used to account for these variables' potential confounding effects on the outcome variables.*

**Response:** Thanks! We have revised this statement: “The analysis included all the available sociodemographic factors in the STEPs datasets from the three countries. The following variables were considered as independent variables/confounders in every model: age group in years (18-29 years, 30-49 years, 50-69 years); gender (male, female); highest educational attainment (no formal schooling, up to primary, up to secondary, college and higher); marital status (never married, currently married, divorced/widowed/separated); smoking status (never smoker, current smoker, former smoker); and ever alcohol consumption (yes, no). For cleaning teeth at least once and at least twice a day, we included ‘previous dental visit’ as a covariate, which was categorized as follows: less than 6 months, 6-12 months, more than 12 months, and never visited (which included more than 1 year but less than 2 years, 2 or more years but less than 5 years; 5 or more years).” Also, multivariable multilevel logistic regressions and multivariable log-binomial model were conducted to account for these variables' potential confounding effects on the outcome variables. We have already mentioned this in the statistical analysis section.

*5. Confounders can distort the true relationship between the independent and dependent variables. The methodology mentions a Directed Acyclic Graph (DAG) showing potential confounding paths between the variables. However, it doesn't explicitly state whether all identified confounders were accounted for in the analysis. The authors could clarify which confounders were considered and how they were addressed in the analysis.*

**Response:** Thanks! We have revised this statement: “The analysis included all the available sociodemographic factors in the STEPs datasets from the three countries. The following variables were considered as independent variables/confounders in every model: age group in years (18-29 years, 30-49 years, 50-69 years); gender (male, female); highest educational attainment (no formal schooling, up to primary, up to secondary, college and higher); marital status (never married, currently married, divorced/widowed/separated); smoking status (never smoker, current smoker, former smoker); and ever alcohol consumption (yes, no). For cleaning teeth at least once and at least twice a day, we included ‘previous dental visit’ as a covariate, which was categorized as follows: less than 6 months, 6-12 months, more than 12 months, and never visited (which included more than 1 year but less than 2 years, 2 or more years but less than 5 years; 5 or more years).”

*6. Standardisation methods can help control for confounding variables in statistical analysis. The methodology doesn't mention specific standardization techniques used to neutralize confounders. The authors need to explicitly clarify if any standardisation or adjustment methods were employed to mitigate confounding effects.*

**Response:** Thanks! Multivariable multilevel logistic regressions and multivariable log-binomial model were conducted to account for these variables' potential confounding effects on the outcome variables. We have already mentioned this in the statistical analysis section.

*7. The background information provided by the authors suggests that adherence to oral hygiene practices has been linked to higher socioeconomic status and education level. However, the specific context of Bangladesh, Bhutan, and Nepal may differ from previous studies, and the authors could consider mentioning that the aim was also to investigate the associations between oral health practices and sociodemographic factors within these countries.*

**Response:** Thanks! We have revised the following: “This study aims to investigate the prevalence of and factors (including the socio-demographic factors) associated with teeth cleaning frequency and dental visits in the past year among residents of these three South Asian countries, using nationally representative data.”
