# Supplementary material for: Prevalence and associated factors of last dental visit and teeth cleaning frequency in Bangladesh, Bhutan, and Nepal: Findings from nationally representative surveys
Source: PLOS Glob Public Health. 2024 Jul 19;4(7):e0003511. doi: 10.1371/journal.pgph.0003511 (PMC11259307; doi:10.1371/journal.pgph.0003511)
Supplement: Attachment — Submitted filename: Plos review 20240520.docx [file pgph.0003511.s021.docx]

1. **Rationale in Introduction**

Authors could strengthen their introduction by considering the following suggestions-

- Briefly mention previous research linking socioeconomic status and education to oral hygiene practices.
- State that **limited research has been conducted in Bangladesh, Bhutan, and Nepal**, and there might be contextual variations influencing these relationships.
- Clearly, mention that one of the aims of the study is to investigate these associations within these specific countries.

By incorporating these points, the introduction would provide a stronger rationale for the research and its potential to contribute new knowledge.

2. **Data Sources:** It is a secondary analysis of existing data, so the researchers might be limited by what information was collected in the original surveys.

**Limitations of Secondary Data Analysis:**

- **Limited Variable Availability:** As this is a secondary analysis, the researchers are restricted by the data collected in the original WHO STEPS surveys. There might be important factors influencing oral health behaviours that were not included in these surveys, and thus cannot be explored in this study. This could be stated as limitation in the discussion.
- **Potential for Bias in Original Data Collection:** The original data collection might have been influenced by factors like participant recall or social desirability bias. This could affect the accuracy of the findings in this secondary analysis.

**Lack of detail about the primary data**

- **Missing Data:** It is not clear if there is any missing data in the original surveys and how the researchers handled it.
- **Power Analysis:** The article does not mention if a power analysis was conducted to determine the sample size needed to detect statistically significant effects. Adequate sample size is crucial for reliable results.

The lack of detail about the primary data collection methods is a valid critique of the methodology section. Please include the following information:

- **Questionnaire Design:** Understanding the questionnaire's structure, wording of questions, and response options can help assess potential biases or limitations in how data was collected. For instance, leading questions or ambiguous answer choices could influence participant responses.
- **Interviewer Training:** Information about the training interviewers received on question interpretation, probing techniques, and maintaining consistency can help ensure data quality. Inconsistent application of interviewing methods could introduce bias.
- **Quality Control Measures:** Knowing the quality control measures used during data collection, such as pilot testing the questionnaire or conducting inter-rater reliability checks for physical measurements, can provide confidence in the accuracy and reliability of the data.

Without details on these aspects, it is challenging to fully evaluate the robustness of the primary data used in the secondary analysis. Some suggestions for authors:

- **References:** The methodology section could mention that the specific methods for the WHO STEPS surveys are described elsewhere.
- **Consider limitations:** When interpreting the results of the study, it is important to acknowledge the limitations arising from the lack of detail about the primary data collection methods. Authors can mention this as a caveat in the analysis

3. **Confounding Variables**

**Confounding Variable:** The introduction acknowledges the bidirectional relationship between dental visits and teeth cleaning frequency. The analysis plan addresses this by including them as covariates in the models. Overall, the methodology suggests the data is likely somewhat representative due to the use of nationally representative surveys. However, the lack of information on stratification criteria and sample size within strata makes it difficult to confirm perfect proportionality. It is crucial to explicitly clarify adjustment methods for confounding variables in a well-designed study for the following reasons-

- **Confounding can introduce bias:** For example, age could confound the relationship between income and dental visits. Older adults might have lower income and fewer dental visits, but this doesn't necessarily mean income directly causes fewer visits.
- **Adjustment Methods Control for Bias:** Statistical methods like regression modelling can be used to statistically "adjust for" the confounding variables. This essentially removes the influence of the confounding variable on the relationship of interest.
- **Unclear about comprehensiveness:** It does not explicitly state whether all identified confounders were included in the analysis. Ideally, the methodology should mention which confounders were considered most relevant and how they were addressed statistically.

But for a more definitive assessment, it would be helpful to see the following in the discussion section of the manuscript:

- **Mentioning limitations of available variables:** Acknowledge that the research was limited by the variables included in the original WHO STEP surveys and could not explore other potentially relevant factors influencing oral health behaviours
- **Addressing potential bias in original data collection:** Discuss the possibility of bias in the original surveys due to participant recall or social desirability, and how this might affect the analysis.

The following suggestions could be considered by the authors to strengthen their methodology:

- **List the considered confounders:** Explicitly state which sociodemographic factors (e.g., age, gender, education) and other variables (e.g., smoking status) were the confounders based on their literature review.
- **Explain adjustment methods:** Clarify how confounders were addressed in the analysis. The methodology mentions multivariable logistic regression and log-binomial models, which are appropriate techniques for controlling confounding variables. However, it would be clearer if the authors explicitly stated this purpose. would strengthen the methodology section.

4. **Proportional data representation**

The methodology **doesn't explicitly mention** whether the data from each country is proportional to its diverse population. However, it describes the sampling strategy used, which can help us understand the representativeness of the data.

- **Nationally Representative Surveys:** The text mentions these are secondary analyses of nationally representative WHO STEPS surveys from Bangladesh, Bhutan, and Nepal. This suggests the surveys aimed to include participants from across each country, increasing the likelihood of capturing some population diversity.

However, there's still a possibility that the data might not be perfectly proportional.

**Multistage Stratified Cluster Sampling:** The methodology describes using a multistage stratified cluster sampling approach. This means the population was divided into subgroups (strata) based on certain characteristics (e.g., urban/rural areas). Then, clusters (e.g., villages or city blocks) were randomly selected within each stratum. While stratification helps account for some population heterogeneity, it might not perfectly capture all subgroups within a stratum if the sample size within each stratum is not proportional to its size in the population.

To definitively assess proportionality, authors could provide the following information-

- **Stratification criteria:** What characteristics were used to divide the population into strata (e.g., urban/rural, age groups)?
- **Sample size within strata:** Was the sample size within each stratum proportionate to the size of that subgroup in the national population?

The provided methodology section mentions including sociodemographic factors like age, gender, and education as independent variables. This suggests that the authors planned to account for these variables in the analysis. While the methodology suggests they will likely address confounding, explicitly mentioning the adjustment method would strengthen the results.

5. **Biochemical measurements involving, urine, blood glucose and lipid assessments**

The revised manuscript mentions the collection of biological samples such as urine, blood glucose and lipids. There is no mention of the rationale for collecting these samples and the corresponding results and the correlation to the oral health behaviours and demographics is not explained. This is confusing and requires clarification.
