# Supplementary material for: Prevalence and associated factors of last dental visit and teeth cleaning frequency in Bangladesh, Bhutan, and Nepal: Findings from nationally representative surveys
Source: PLOS Glob Public Health. 2024 Jul 19;4(7):e0003511. doi: 10.1371/journal.pgph.0003511 (PMC11259307; doi:10.1371/journal.pgph.0003511)
Supplement: Attachment — Submitted filename: Response to reviewer.docx [file pgph.0003511.s022.docx]

**Manuscript title: Prevalence and associated factors of last dental visit and teeth cleaning frequency in Bangladesh, Bhutan, and Nepal: Findings from nationally representative surveys.**

**Manuscript ID: PGPH-D-24-00051R2**

**General comments from authors:** We are grateful to the reviewers for their kind comments and valuable time towards the progress of our manuscript. We have added our step-by-step specific responses and pasted the additional sentences from the revised manuscript in blue fonts.

1. ***Rationale in Introduction***

*Authors could strengthen their introduction by considering the following suggestions-*

- *Briefly mention previous research linking socioeconomic status and education to oral hygiene practices.*

Response to the reviewer: Thanks! We have added the following paragraph:

“Previous research has consistently demonstrated a strong association between socioeconomic status (SES) and education to oral hygiene practices. Studies have shown that individuals with higher SES and educational attainment are more likely to engage in regular oral hygiene behaviors, such as brushing and flossing, and are more likely to visit dental care professionals regularly. Conversely, those with lower SES and less education often have poorer oral hygiene practices and are at greater risk for dental diseases. These disparities can be attributed to differences in access to dental care, health literacy, and overall health behaviors influenced by socioeconomic and educational factors [26–30].”

- *State that* ***limited research has been conducted in Bangladesh, Bhutan, and Nepal****, and there might be contextual variations influencing these relationships.*

**Response to the reviewer:** Thanks! We have added the following paragraph: “Despite the extensive research linking sociodemographic factors to oral hygiene practices globally, few studies on oral hygiene practices and related sociodemographic factors are available for residents of Bangladesh, Nepal, and Bhutan [29,30]. Moreover, these studies have not utilized nationally representative data. These countries present unique contextual variations that may influence the relationship between socioeconomic factors and oral hygiene behaviors. Cultural, economic, and healthcare system differences in these regions could result in distinct patterns and determinants of oral health practices not fully captured by studies conducted in other contexts. Therefore, it is crucial to investigate these relationships within these specific countries to develop targeted and effective public health interventions.”

- *Clearly, mention that one of the aims of the study is to investigate these associations within these specific countries.*

**Response to the reviewer:** Thanks! We have already added the following sentence: “This study aims to investigate the prevalence of and factors (including the socio-demographic factors) associated with teeth cleaning frequency and dental visits in the past year among residents of these three South Asian countries (Bangladesh, Bhutan and Nepal), using nationally representative data.”

*By incorporating these points, the introduction would provide a stronger rationale for the research and its potential to contribute new knowledge.*

**Response to the reviewer:** Thanks! We have incorporated the comments.

2. ***Data Sources:*** *It is a secondary analysis of existing data, so the researchers might be limited by what information was collected in the original surveys.*

***Limitations of Secondary Data Analysis:***

- ***Limited Variable Availability:*** *As this is a secondary analysis, the researchers are restricted by the data collected in the original WHO STEPS surveys. There might be important factors influencing oral health behaviours that were not included in these surveys, and thus cannot be explored in this study. This could be stated as limitation in the discussion.*

**Response to the reviewer:** Thanks! We have added it in the limitation section: “As this is a secondary analysis, the researchers are restricted by the data collected in the original WHO STEPS surveys. There might be important factors influencing oral health behaviours that were not included in these surveys, and thus cannot be explored in this study. For instance, the WHO STEPS datasets did not contain information on wealth status, preventing adjustment for this variable in our analysis. Due to data limitations, Urban/rural differences could not be explored in Nepal.

Additionally, the lack of comprehensive oral health data in other South Asian nations prevented regional comparisons. Our oral health outcomes were limited to only two main variables, as no other commonly measured oral health outcomes were consistent across the three surveys. This study utilized secondary datasets, which provide valuable insights through extensive coverage and standardized methodologies but is limited by data quality variability and the potential misalignment of variables with our specific research objectives.”

- ***Potential for Bias in Original Data Collection:*** *The original data collection might have been influenced by factors like participant recall or social desirability bias. This could affect the accuracy of the findings in this secondary analysis.*

**Response to the reviewer:** Thanks! We have added this in the limitation section: “The original data collection may have been impacted by factors such as participant recall bias or social desirability bias. These influences could compromise the accuracy of the findings in this secondary analysis.”

***Lack of detail about the primary data***

- ***Missing Data:*** *It is not clear if there is any missing data in the original surveys and how the researchers handled it.*

**Response to the reviewer:** Thanks! We have added in the statistical analysis section “Complete case analysis was conducted given the very low percentage of missing data (<1%) [37].”

- ***Power Analysis:*** *The article does not mention if a power analysis was conducted to determine the sample size needed to detect statistically significant effects. Adequate sample size is crucial for reliable results.*

**Response to the reviewer:** Thank you for your insightful feedback regarding the power analysis.

Our study utilized nationally representative samples from the WHO STEPwise approach to surveillance (STEPS) surveys conducted in Bangladesh, Bhutan, and Nepal. These datasets are substantial and meticulously designed to detect statistically significant effects for all the collected variables, including those used in our study.

The sample sizes for these surveys were rigorously calculated to ensure adequate power to detect meaningful associations and effects. The comprehensive methodology, including sample size calculations, has been thoroughly documented and cited in the original reports. As noted in our manuscript, "The surveys' methods, sampling strategy, sample size calculation, data collection procedure, and preliminary results have been published elsewhere [18,31,32]."

We believe that the robust design and large sample sizes of these surveys provide a strong foundation for the reliability and validity of our findings.

*The lack of detail about the primary data collection methods is a valid critique of the methodology section. Please include the following information:*

- ***Questionnaire Design:*** *Understanding the questionnaire's structure, wording of questions, and response options can help assess potential biases or limitations in how data was collected. For instance, leading questions or ambiguous answer choices could influence participant responses.*

**Response to the reviewer:** Thank you for your valuable feedback on our study.

Regarding the questionnaire design, we would like to emphasize that the surveys used in our study followed the standardized WHO STEPwise approach to surveillance (STEPS) methodology. This methodology ensures a rigorous and systematic process for questionnaire development, including the structure, wording of questions, and response options.

The questionnaires were carefully designed to avoid leading questions and ambiguous answer choices, minimizing potential participant responses biases. Each question was crafted to be clear and straightforward, ensuring that participants could understand and respond accurately without undue influence.

The comprehensive process for developing these surveys, including the design and validation of the questionnaires, has been documented and cited in our manuscript. As stated, "The surveys' methods, sampling strategy, sample size calculation, data collection procedure, and preliminary results have been published elsewhere [18,31,32]."

- ***Interviewer Training:*** *Information about the training interviewers received on question interpretation, probing techniques, and maintaining consistency can help ensure data quality. Inconsistent application of interviewing methods could introduce bias.*

**Response to the reviewer:** Thank you for your insightful feedback regarding interviewer training. We would like to provide additional clarification about the training process for interviewers involved in data collection. As mentioned in our manuscript:

"Data collection across three countries employed the WHO NCD STEPS instrument, incorporating three steps with core, expanded, and country-specific questions to measure NCD risk factors. In Bangladesh, the survey included essential and selected optional modules such as oral health and cervical cancer screening and was translated into the local language with subsequent validation through back translation [18,31,32]. Trained enumerators and supervisors who had undergone extensive training on interviewing techniques, household selection, and data recording on Android tablets also received practical training on physical and biochemical measurements. Mock interviews were conducted in local dialects to ensure the quality and standardization of the process. Supervisors specifically focused on overseeing the data collection, ensuring data accuracy, and providing necessary support [18,31,32]."

To elaborate, the training program for interviewers was comprehensive and included:

1. **Question Interpretation and Probing Techniques:** Interviewers were extensively trained on the interpretation of each question to ensure uniform understanding and delivery. Probing techniques were taught to gather complete and accurate responses without leading the participants.
2. **Consistency in Application:** Practical sessions and mock interviews in local dialects were conducted to ensure that interviewers could apply their training consistently across different regions and contexts. This also helped to familiarize them with the survey tools and techniques in a controlled setting.
3. **Supervisory Oversight:** Supervisors played a critical role in maintaining data quality by overseeing the data collection process, ensuring the accuracy of recorded data, and providing immediate support and corrective feedback to interviewers as needed.

The structured training program aimed to minimize any potential biases and ensure the collection of high-quality data across the three countries. We believe that these measures contributed significantly to the robustness and reliability of our study findings.

- ***Quality Control Measures:*** *Knowing the quality control measures used during data collection, such as pilot testing the questionnaire or conducting inter-rater reliability checks for physical measurements, can provide confidence in the accuracy and reliability of the data.*

**Response to the reviewer:** Thank you for your valuable feedback regarding the quality control measures used during data collection. We would like to provide further details on the quality control measures implemented to ensure the accuracy and reliability of the data. As mentioned in our manuscript:

"Data collection across three countries employed the WHO NCD STEPS instrument, incorporating three steps with core, expanded, and country-specific questions to measure NCD risk factors. In Bangladesh, the survey included essential and selected optional modules such as oral health and cervical cancer screening. It was translated into the local language with subsequent validation through back translation [18,31,32]. Trained enumerators and supervisors, who had undergone extensive training on interviewing techniques, household selection, and data recording on Android tablets, also received practical training on physical and biochemical measurements. Mock interviews were conducted in local dialects to ensure the quality and standardization of the process. Supervisors specifically focused on overseeing the data collection, ensuring data accuracy, and providing necessary support [18,31,32]."

To further elaborate, the following quality control measures were undertaken:

1. **Pilot Testing:** Prior to the main data collection, the questionnaire underwent pilot testing to identify and rectify any potential issues in question interpretation, response options, and overall survey flow. This step was crucial in ensuring that the final questionnaire was clear, unambiguous, and effective in capturing the required data.
2. **Regular Supervision and Monitoring:** Supervisors closely monitored the data collection process, conducting regular checks to ensure adherence to protocols. They provided immediate feedback and support to enumerators, addressing any issues promptly to maintain data quality.
3. **Data Validation and Cleaning:** Post-collection, the data underwent rigorous validation and cleaning processes. This included cross-checking entries, verifying outliers, and ensuring completeness and accuracy of the data before analysis.
4. **Use of Technology:** The use of Android tablets for data recording helped minimize manual errors and facilitated real-time data validation. This technological integration ensured that data was recorded accurately and efficiently.

These quality control measures were integral to the data collection process, ensuring the accuracy, reliability, and validity of the data collected across the three countries. We believe that these measures significantly enhanced the robustness of our study findings.

*Without details on these aspects, it is challenging to fully evaluate the robustness of the primary data used in the secondary analysis. Some suggestions for authors:*

- ***References:*** *The methodology section could mention that the specific methods for the WHO STEPS surveys are described elsewhere.*

**Response to the reviewer:** Thank you for this comment. We have mentioned in the methods section the following: "The surveys' methods, sampling strategy, sample size calculation, data collection procedure, and preliminary results have been published elsewhere [18,31,32]."

- ***Consider limitations:*** *When interpreting the results of the study, it is important to acknowledge the limitations arising from the lack of detail about the primary data collection methods. Authors can mention this as a caveat in the analysis*

**Response to the reviewer:** We have mentioned “The study has limitations, including its cross-sectional nature, which precludes causal inference, and a limited number of correlates in the sample . When interpreting the results of this study, it is important to acknowledge the limitations arising from the lack of detailed information about the primary data collection methods. This caveat should be considered when assessing the robustness of our findings. As this is a secondary analysis, the researchers are restricted by the data collected in the original WHO STEPS surveys. There might be important factors influencing oral health behaviours that were not included in these surveys, and thus cannot be explored in this study. For instance, the WHO STEPS datasets did not contain information on wealth status, preventing adjustment for this variable in our analysis. Urban/rural differences could not be explored in Nepal due to data limitations. Additionally, the lack of comprehensive oral health data in other South Asian nations prevented regional comparisons . Our oral health outcomes were limited to only two main variables, as no other commonly measured oral health outcomes were consistent across the three surveys. This study utilized secondary datasets, which provide valuable insights through extensive coverage and standardized methodologies but is limited by data quality variability and the potential misalignment of variables with our specific research objectives. The original data collection may have been impacted by factors such as participant recall bias or social desirability bias. These influences could compromise the accuracy of the findings in this secondary analysis. However, the study's strengths include the use of nationally representative data and validated questionnaires, reducing the likelihood of information bias.”

3. ***Confounding Variables***

***Confounding Variable:*** *The introduction acknowledges the bidirectional relationship between dental visits and teeth cleaning frequency. The analysis plan addresses this by including them as covariates in the models. Overall,* *the methodology suggests the data is likely somewhat representative due to the use of nationally representative surveys. However, the lack of information on stratification criteria and sample size within strata makes it difficult to confirm perfect proportionality. It is crucial to explicitly clarify adjustment methods for confounding variables in a well-designed study for the following reasons-*

- ***Confounding can introduce bias:*** *For example, age could confound the relationship between income and dental visits. Older adults might have lower income and fewer dental visits, but this doesn't necessarily mean income directly causes fewer visits.*
- ***Adjustment Methods Control for Bias:*** *Statistical methods like regression modelling can be used to statistically "adjust for" the confounding variables. This essentially removes the influence of the confounding variable on the relationship of interest.*
- ***Unclear about comprehensiveness:*** *It does not explicitly state whether all identified confounders were included in the analysis. Ideally, the methodology should mention which confounders were considered most relevant and how they were addressed statistically.*

**Response to the reviewer:** Thank you for this comment. Multivariable multilevel logistic regressions were used to adjust the confounding. For sensitivity analyses, we used multivariable log-binomial model to adjust the confounding and compare the findings with the multivariable multilevel logistic regressions model. We have revised the statistical analysis as following: “Bivariate and multivariable multilevel logistic regressions were performed to identify the factors associated with oral hygiene practices. These analyses included adjustments for sociodemographic factors such as age, gender, and education to account for potential confounding variables. By adjusting for these factors, we aimed to ensure the robustness and reliability of our results, providing a more accurate understanding of the associations between the independent variables and oral hygiene practices. We reported both the crude odds ratio (COR) and the adjusted odds ratio (AOR), along with a 95% confidence interval (CI). We also conducted a log-binomial model and reported crude prevalence ratio (CPR) for the unadjusted model and adjusted prevalence ratio (APR) for the multivariable log-binomial model. 95% CI was reported for both CPR and APR.”

*But for a more definitive assessment, it would be helpful to see the following in the discussion section of the manuscript:*

- ***Mentioning limitations of available variables:*** *Acknowledge that the research was limited by the variables included in the original WHO STEP surveys and could not explore other potentially relevant factors influencing oral health behaviours*

**Response to the reviewer:** Thanks! We have revised the limitation section as following: “The study has limitations, including its cross-sectional nature, which precludes causal inference, and a limited number of correlates in the sample. As this is a secondary analysis, the researchers are restricted by the data collected in the original WHO STEPS surveys. There might be important factors influencing oral health behaviours that were not included in these surveys, and thus cannot be explored in this study. For instance, the WHO STEPS datasets did not contain information on wealth status, preventing adjustment for this variable in our analysis. Urban/rural differences could not be explored in Nepal due to data limitations. Additionally, the lack of comprehensive oral health data in other South Asian nations prevented regional comparisons.”

- **Addressing potential bias in original data collection:** Discuss the possibility of bias in the original surveys due to participant recall or social desirability, and how this might affect the analysis.

**Response to the reviewer:** Thanks! We have revised the limitation section as following: “The original data collection may have been impacted by factors such as participant recall bias or social desirability bias. These influences could compromise the accuracy of the findings in this secondary analysis.”

*The following suggestions could be considered by the authors to strengthen their methodology:*

- ***List the considered confounders:*** *Explicitly state which sociodemographic factors (e.g., age, gender, education) and other variables (e.g., smoking status) were the confounders based on their literature review.*

**Response to the reviewer:** Thank you for the kind comment. We have already addressed this concern in the methods section as following : “The analysis included all the available sociodemographic factors in the STEPs datasets from the three countries. The following variables were considered as independent variables/confounders in every model: age group in years (18-29 years, 30-49 years, 50-69 years); gender (male, female); highest educational attainment (no formal schooling, up to primary, up to secondary, college and higher); marital status (never married, currently married, divorced/widowed/separated); smoking status (never smoker, current smoker, former smoker); and ever alcohol consumption (yes, no). For cleaning teeth at least once and at least twice a day, we included ‘previous dental visit’ as a covariate, which was categorized as follows: less than 6 months, 6-12 months, more than 12 months, and never visited (which included more than 1 year but less than 2 years, 2 or more years but less than 5 years; 5 or more years). For visiting a dentist within the last 6 or 12 months or never visiting a dentist, we used teeth cleaning frequency as a covariate, which was categorized into once a day, twice a day, and infrequent/never (which included never; once a month; 2-3 times a month; once a week; 2-6 times a week). Smoking within the 30 days before data collection was defined as current smoking [18,31,32]. The relationship between the variables is presented in a directed acyclic graph (DAG) in Figure 1. The DAG shows a bidirectional relationship between the last dental visit and the frequency of teeth cleaning. The paths that are marked as red imply confounding paths, which needed to be adjusted during the analysis.”

- ***Explain adjustment methods:*** *Clarify how confounders were addressed in the analysis. The methodology mentions multivariable logistic regression and log-binomial models, which are appropriate techniques for controlling confounding variables. However, it would be clearer if the authors explicitly stated this purpose. would strengthen the methodology section.*

**Response to the reviewer:** Thank you for this comment. Multivariable multilevel logistic regressions were used to adjust the confounding. For sensitivity analyses, we used multivariable log-binomial model to adjust the confounding and compare the findings with the multivariable multilevel logistic regressions model. We have revised the statistical analysis as following: “Bivariate and multivariable multilevel logistic regressions were performed to identify the factors associated with oral hygiene practices. These analyses included adjustments for sociodemographic factors such as age, gender, and education to account for potential confounding variables. By adjusting for these factors, we aimed to ensure the robustness and reliability of our results, providing a more accurate understanding of the associations between the independent variables and oral hygiene practices. We reported both the crude odds ratio (COR) and the adjusted odds ratio (AOR), along with a 95% confidence interval (CI). We also conducted a log-binomial model and reported crude prevalence ratio (CPR) for the unadjusted model and adjusted prevalence ratio (APR) for the multivariable log-binomial model. 95% CI was reported for both CPR and APR.”

4. **Proportional data representation**

*The methodology* ***doesn't explicitly mention*** *whether the data from each country is proportional to its diverse population. However, it describes the sampling strategy used, which can help us understand the representativeness of the data.*

- ***Nationally Representative Surveys:*** *The text mentions these are secondary analyses of nationally representative WHO STEPS surveys from Bangladesh, Bhutan, and Nepal. This suggests the surveys aimed to include participants from across each country, increasing the likelihood of capturing some population diversity.*

*However, there's still a possibility that the data might not be perfectly proportional.*

***Multistage Stratified Cluster Sampling:*** *The methodology describes using a multistage stratified cluster sampling approach. This means the population was divided into subgroups (strata) based on certain characteristics (e.g., urban/rural areas). Then, clusters (e.g., villages or city blocks) were randomly selected within each stratum. While stratification helps account for some population heterogeneity, it might not perfectly capture all subgroups within a stratum if the sample size within each stratum is not proportional to its size in the population.*

*To definitively assess proportionality, authors could provide the following information-*

- ***Stratification criteria:*** *What characteristics were used to divide the population into strata (e.g., urban/rural, age groups)?*
- ***Sample size within strata:*** *Was the sample size within each stratum proportionate to the size of that subgroup in the national population?*

**Response to the reviewer:** Thanks! Thank you for your comments. The population was divided into strata based on urban and rural area and the sample size within each stratum was proportionate to the size of that subgroup in the national population We have revised the methodology section to include the requested details about the stratification criteria and sample size proportionality: “A multistage stratified cluster sampling of households was employed in each country. The population was divided into strata based on urban and rural areas. Random selections of primary sampling units (PSUs) were made from both urban and rural areas. In all three countries, the PSUs were selected based on probability proportional to size methods. The sample size within each stratum was proportionate to the size of that subgroup in the national population, ensuring a representative sample. Participants were chosen from these selected households.”

*The provided methodology section mentions including sociodemographic factors like age, gender, and education as independent variables. This suggests that the authors planned to account for these variables in the analysis. While the methodology suggests they will likely address confounding, explicitly mentioning the adjustment method would strengthen the results.*

**Response to the reviewer:** Thanks! We have already mentioned that “Bivariate and multivariable multilevel logistic regressions were performed to identify the factors associated with oral hygiene practices.” Additionally, we want to clarify that these analyses included adjustments for sociodemographic factors such as age, gender, and education to account for potential confounding variables. This adjustment ensures the robustness and reliability of our results. We have revised the stated section as follows:

“Bivariate and multivariable multilevel logistic regressions were performed to identify the factors associated with oral hygiene practices. These analyses included adjustments for sociodemographic factors such as age, gender, and education to account for potential confounding variables. By adjusting for these factors, we aimed to ensure the robustness and reliability of our results, providing a more accurate understanding of the associations between the independent variables and oral hygiene practices.”

5. ***Biochemical measurements involving, urine, blood glucose and lipid assessments***

*The revised manuscript mentions the collection of biological samples such as urine, blood glucose and lipids. There is no mention of the rationale for collecting these samples and the corresponding results and the correlation to the oral health behaviours and demographics is not explained. This is confusing and requires clarification.*

**Response to the reviewer:** Thank you for your feedback. We have revised the statement accordingly: “Additionally, participants were given an appointment card detailing fasting instructions for subsequent biochemical measurements involving blood glucose and lipid assessments. However, this study did not include any biochemical measurements.”
